# The making of an octopus arm

**DOI:** 10.1186/s13227-015-0012-8

**Published:** 2015-05-07

**Authors:** Marie-Therese Nödl, Sara M Fossati, Pedro Domingues, Francisco J Sánchez, Letizia Zullo

**Affiliations:** Department of Neuroscience and Brain Technologies, Istituto Italiano di Tecnologia, Via Morego 30, 16163 Genoa, Italy; Centro Oceanografico de Vigo, Instituto Español de Oceanografia, Subida Radio Faro, 50 36390 Vigo, Spain

**Keywords:** Cephalopod, Octopus, Lophotrochozoa, Appendage development, Muscle development, Evolution, Epithelial cell dynamics, Actin, Myosin heavy chain, Tropomyosin

## Abstract

**Background:**

Most of our current findings on appendage formation and patterning stem from studies on chordate and ecdysozoan model organisms. However, in order to fully understand the evolution of animal appendages, it is essential to include information on appendage development from lophotrochozoan representatives. Here, we examined the basic dynamics of the *Octopus vulgaris* arm’s formation and differentiation - as a highly evolved member of the lophotrochozoan super phylum - with a special focus on the formation of the arm’s musculature.

**Results:**

The octopus arm forms during distinct phases, including an early outgrowth from an epithelial thickening, an elongation, and a late differentiation into mature tissue types. During early arm outgrowth, uniform proliferation leads to the formation of a rounded bulge, which subsequently elongates along its proximal-distal axis by means of actin-mediated epithelial cell changes. Further differentiation of all tissue layers is initiated but end-differentiation is postponed to post-hatching stages. Interestingly, muscle differentiation shows temporal differences in the formation of distinct muscle layers. Particularly, first myocytes appear in the area of the future transverse prior to the longitudinal muscle layer, even though the latter represents the more dominant muscle type at hatching stage. Sucker rudiments appear as small epithelial outgrowths with a mesodermal and ectodermal component on the oral part of the arm. During late differentiation stages, cell proliferation becomes localized to a distal arm region termed the growth zone of the arm.

**Conclusions:**

*O. vulgaris* arm formation shows both, similarities to known model species as well as species-specific patterns of arm formation. Similarities include early uniform cell proliferation and actin-mediated cell dynamics, which lead to an elongation along the proximal-distal axis. Furthermore, the switch to an adult-like progressive distal growth mode during late differentiation stages is reminiscent of the vertebrate progress zone. However, tissue differentiation shows a species-specific delay, which is correlated to a paralarval pelagic phase after hatching and concomitant emerging behavioral modifications. By understanding the general dynamics of octopus arm formation, we established a basis for further studies on appendage patterning, growth, and differentiation in a representative of the lophotrochozoan super phylum.

**Electronic supplementary material:**

The online version of this article (doi:10.1186/s13227-015-0012-8) contains supplementary material, which is available to authorized users.

## Background

The evolution of animal appendages and their convergence or homology on a structural and genetic level has been a topic of ongoing debate over the past century. Most of what we know about the early outgrowth and differentiation of these structures derived from studies on ecdysozoan and chordate model organisms, which revealed striking similarities in the gene regulatory networks involved in early appendage formation and patterning (reviewed in [1-4]).

In particular, within the ecdysozoa, the fruitfly *Drosophila melanogaster* has pioneered the studies on appendage formation. Adult *Drosophila* appendages originate from imaginal discs, which arise as invaginations of the embryonic epidermis. These structures increase in size through cell proliferation and elongate at the end of larval development to form adult appendages (reviewed in [5,6]). Furthermore, within the chordates, vertebrate limb formation has become an important model for studying pattern formation during embryonic development. Vertebrate limbs are initiated by the migration of mesenchymal cells from the lateral plate mesoderm and the lateral dermomyotome into the prospective limb fields. These cells proliferate while the appendage elongates along its proximal-distal axis to form a paddle-shaped limb bud. Two important organizing structures at the distal tip of the arm, the apical ectodermal ridge (AER), and the zone of polarizing activity (ZPA) are coordinating axial patterning, outgrowth, and differentiation of the limb (reviewed in [7-11]). In addition to the studies in established model organisms, a fair amount of comparative data is available on appendage formation and patterning in a wide range of ecdysozoan and chordate phyla (for example, [12-22]). However, only a few studies have addressed this issue in lophotrochozoa [23-26]. Yet, studying the formation and differentiation of appendages in representatives of all super phyla is essential for extending our understanding of appendage evolution.

Modern cephalopods offer a rare opportunity to study the formation of appendages within the lophotrochozoa. Their arm crown is thought to be derived from the molluskan ventral foot and constitutes a morphological novelty, since no comparable structure exists within the phylum [23,27]. The bilaterally symmetrical arm crown consists of four pairs of prehensile and amenable arms encircling the central mouth with an additional pair of retractile tentacles in the decabrachian cephalopods. However, the octopus arm crown seems to have lost these feeding tentacles and is thus regarded as a derived version thereof [28-30].

**Figure 1:**
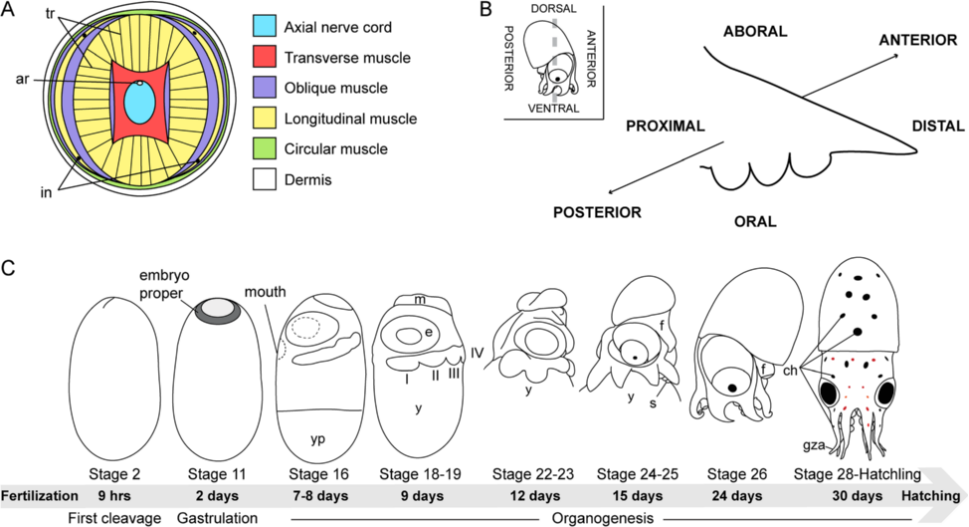
Schematic representation of the octopus arm anatomy and embryonic development. **(A)** Schematic of a cross section through an adult *O. vulgaris* arm. **(B)** Sketch drawing of an octopus hatchling’s arm, demonstrating the arm axes and terminology used in this study. Inset on the top left illustrates the embryonic body axes with respect to the arm’s axes. **(C)** Illustration of selected developmental stages highlighting the major events of the embryonic development from fertilization to hatching. Embryos are shown with anterior to the left and dorsal to the top, except for the hatchling, which is shown in a frontal view. Dark gray shading in stage 11 represents the embryo proper during gastrulation; light gray shading highlights a single cell layer of the not yet gastrulated area. Embryos stages 22 to 26 are displayed without yolk sac. Abbreviations: ar, artery; ch, chromatophore; e, eye; f, funnel; gza, growth zone of the arm; m, mantel; s, sucker; tr, trabeculae; in, intramuscular nerve; y, yolk; yp, yolk plug; I-IV, arm numbers I to IV.

One of the most intriguing features of the cephalopod arm is the three-dimensional combination of longitudinally, transversely, and obliquely arranged muscle fibers and connective tissue. This structure was termed a muscular hydrostat by Kier and Smith [31] because it both provides the arm with skeletal support and allows the animal to perform complex motor activities. The latter are controlled by an axial nerve cord consisting of a series of ganglia arranged along a pair of nerve bundles and a dense peripheral nerve net, which locally perceives and possibly even processes part of the sensory input [32,33] (Figure 1A,B). Each of the ganglia within the axial nerve cord corresponds to one sucker situated on the oral side of the arm. In the octopod cephalopod *Octopus vulgaris*, these suckers cover the arm in a double-row and in addition to the general function of the arm aid in grooming, mating behavior, fine manipulation, and chemotactile recognition [34-36]. All these features contribute to a highly flexible and adaptable structure, which allows the animal to perform a wide range of complex behavioral tasks including prey capture and handling, object manipulation, locomotion, and copulation.

Furthermore, the octopus arm in particular maintains the ability of indeterminate, life-long growth throughout adult ontogeny [37] and possesses high regenerative capabilities [38,39]. Even though the morphological aspects of octopus arm regeneration have been described, we are only in the early stages of understanding some of the molecular components involved in the reestablishment of a morphologically normal and functional adult arm [40]. Likewise, even though comparative studies on the cephalopod arm crown development and evolution exist (for example, [27-29,41-43]), surprisingly little is known about the general dynamics of arm formation and differentiation on a tissue level [44]. Specifically, no morphological or genetic studies have addressed the early formation of the octopus arm’s three-dimensional muscle complex so far.

In this study, we provide a detailed description of the tissue dynamics during the embryonic formation and differentiation of the *O. vulgaris* arm with a special focus on the differentiation of the arm’s musculature. We show that octopus appendage formation can be divided into three distinct phases, each one featuring a specific developmental event. During a first phase of arm outgrowth, strong cell proliferation leads to a formation of a spherical bulge, which in the subsequent phase of elongation extends along its proximal-distal axis. We show that the elongation of this spherical bulge is mediated by epithelial cell shape changes, which rely on actin polymerization during a specific developmental time window. We further indicate that while the differentiation of muscle cells is initiated after an early arm bulge has formed, first muscle layers appear only during the differentiation phase of the arm. Furthermore, after an initial establishment of the embryonic outline, the growth dynamic of the arm switches to an adult-like distal outgrowth. Our study demonstrates parallels in the early formation of the arm to known model organisms as well as species-specific pattern paralleling the animal’s lifestyle in the late developmental stages. We thus establish a basis for further studies on appendage formation and the gene regulatory networks involved within a representative of the lophotrochozoan super phylum.

## Methods

### Animal collection and fixation

Live egg strings of *O. vulgaris* embryos were obtained from the Instituto Español de Oceanografía, Centro Oceanográfico de Vigo. Embryos were kept in 120 × 50 × 45 cm size marine aquaria in well-aerated artificial seawater (ASW) at 18°C. Animals were staged according to [45], dissected manually from the protective egg envelope, relaxed in 3.7% MgCl_2_ in ASW, and fixed in 4% paraformaldehyde (PFA) in ASW. Specimen were either dehydrated in a graded ethanol or methanol series and stored in 70% ethanol at 4°C or 100% methanol at −80°C, or stored in 1× PBS at 4°C until further processing. All experiments involving live embryos were performed before March 2014, when the European directive 86/609/EEC with the D.Lgs.n. 26/2014 came into force in Italy, which is why no ethical approval was requested.

However, our research conformed to the ethical principles of replacement, reduction, refinement, and minimization of animal suffering [46] following the guidelines reported in the European directive 86/609/EEC (D.Lgs.n. 26/2014). Particular attention was given to the method of housing, animal care, and health monitoring as well as to identifying signs of pain or distress in the animals.

### Histology

Specimens stored in 70% ethanol were dehydrated in a graded ethanol series and embedded for serial sectioning. Early yolk - rich stages (stages 19 to 24) were embedded in epoxy resin (Epon 812, TAAB Ltd., Rome, Italy) and sectioned into 2-μm semi-thin serial sections using a glass knife and a Leica EM UC6 microtome (Leica Microsystems, Milan, Italy) in order to prevent breaking of the brittle tissue. Sections were stained with toluidine blue for 20 s at 100°C. Later stages (stages 26 to 30) were embedded in paraffin (stages 26 to 30), sectioned into 4-μm thin sections using a Leica RM2125 microtome (Leica Microsystems, Milan, Italy) and stained with Masson’s trichrome stain in order to identify mature muscle fibers. For each stage four embryos were embedded, sectioned, and analyzed.

### Whole-mount antibody/phallacidin staining

Samples were rinsed 3 times for 5 min. in 1× PBS, permeabilized in 1× PBS + 1% Triton X-100 (PBT) for 2 h at room temperature (RT) or overnight at 4°C. Animals were incubated in block solution (PBT + 10% heat-inactivated goat serum (Vector Labs Inc., Segrate, Italy)) for 2 h at RT, and the primary antibody in block solution was applied overnight at 4°C. In order to control for unspecific staining, negative controls were incubated in block solution without the antibody. After several PBS washes, specimen were incubated in secondary antibody, BODIPY FL-phallacidin (F-actin; 1:200; Life Technologies, Milan, Italy), and TO-PRO-3 (nuclei; 1:1,000; Life Technologies, Milan, Italy) overnight at 4°C. Embryos were rinsed several times over a period of 2 h, cleared, and mounted in VECTASHIELD mounting medium (H1000, Vector Laboratories, Segrate, Italy) for analysis. Primary antibodies were used as follows: 1:1000 anti-acetylated tubulin (nerve tracts; clone 6-11B-1; Sigma-Aldrich, Milan, Italy) and 1:200 proliferating cell nuclear antigen (PCNA; ab29, Abcam, Milan, Italy). All antibody/phallacidin stainings were performed on ten individuals per stage and replicated four independent times for each staining type.

### Cytochalasin D treatment

Experiments were conducted in 250-mL sterilized Duran beakers at 18°C. Individual egg strings containing 25 to 40 embryos each were tied to a string and attached to the beaker with adhesive tape. Each beaker was individually aerated and covered by parafilm in order to avoid evaporation of water and changes in salinity. Beakers and solutions were changed every other day. Cytochalasin D (Cyt D; Sigma-Aldrich, Milan, Italy) was applied in 0.05% dimethyl sulfoxide (DMSO, Sigma-Aldrich, Milan, Italy) in ASW containing 25 U penicillin-streptomycin (Pen-Strep; Sigma-Aldrich, Milan, Italy) at concentrations of 1 and 5 μM. Control animals were incubated in ASW containing 25 U Pen-Strep and 0.05% DMSO. Treatment started at stage 18 and was stopped when control embryos had reached stage 23. In total, two independent treatments were performed for each concentration. At stage 23, half of the embryos for each condition were fixed and stained for BODIPY FL-phallacidin and TO-PRO-3 as described above. Cell nuclei of single arms in each condition (control, *n* = 3; embryos treated with 1 μM, *n* = 3; embryos treated with 5 μM, *n* = 4) were counted manually using the ImageJ [47] cell counter tool. Length to width ratio of epithelial cells in each condition (control, *n* = 20; embryos treated with 1 μM, *n* = 20; embryos treated with 5 μM, *n* = 20) was measured using the Adobe Photoshop (Adobe Systems Incorporated, San Jose, CA, USA) ruler tool. The remaining embryos were washed with frequent changes of ASW for 2 h and left to develop in ASW containing 25 U Pen-Strep until the control embryos had reached stage 25. Embryos were then fixed and analyzed as previously described. Length, width, and thickness of single arms in each condition (stage 23: control, *n* = 6; embryos treated with 1 μM, *n* = 7; embryos treated with 5 μM, *n* = 14; stage 25: control, *n* = 4; embryos treated with 1 μM, *n* = 11) were measured using the Adobe Photoshop (Adobe Systems Incorporated, San Jose, CA, USA) ruler tool, measuring pixel length within the acquired confocal images.

### Statistical analysis

Statistical analysis was carried out using SigmaPlot 12.5 (Systat Software, Inc.). The distribution of the datasets was first assessed with the normality test (Shapiro-Wilk). Comparison between two groups was performed with the *t* test or with the Mann-Whitney rank sum test. Pairwise multiple comparisons and comparison *versus* control was performed with the ANOVA test using the Holm-Sidak method. *P* values <0.05 were considered significant.

### Dissociation of muscle cells and cell culture

*O. vulgaris* embryos (stage 30) were anesthetized in 3.7% MgCl_2_ in ASW for 20 min. Animals were decapitated, arm crowns of about 50 animals were dissected manually, and the epithelium was removed. After a 5-min treatment with oxygenated water at 10°C, tissue was incubated for 3 h in 0.2% collagenase (Sigma-Aldrich, Milan, Italy) in L15 medium (Gibco Life Technologies, Milan, Italy) adjusted to seawater concentration (L15-SW). Tissue was rinsed once in L15-SW, vortexed for 3 min and centrifuged for 30 s at maximum speed (14,800 rpm). Supernatant was removed and pellet was resuspended in L15-SW. Concentrations of 20,000 to 100,000 cells were plated on polylysine-covered cover slips and incubated in L15-SW at room temperature over night. Cells were fixed with 4% PFA in artificial seawater for 30 min at RT, washed 3 times for 5 min in PBS, and stained with partition BO-DIPY FL-phallacidin for 1 h at room temperature. Cells were rinsed several times in PBS and mounted in VECTASHIELD mounting medium with partition 4′,6-diamidino-2-phenyl-indole (DAPI) (Vector Laboratories, Segrate, Italy).

### Gene isolation

Total RNA was extracted from a pooled sample of embryos (stages 10 to 30) using the RNeasy Microarray Tissue Mini Kit (Qiagen, Milan, Italy) and retrotranscribed using the ImProm-II™ Reverse Transcription System (Promega, Milan, Italy) following the manufacturers’ protocols. Gene-specific primers for *Ov-Actin* and *Ov-Tropomyosin* (*Ov-Tm*) were designed from the sequences published on GenBank at the NCBI (http://www.ncbi.nlm.nih.gov) with the accession numbers FJ611947.1 and AB218917.1, respectively. For the isolation of the *Ov-Myosin heavy chain* (*Ov-Mhc*) gene fragments, a combination of gene-specific primers designed from the *Loligo pealei-Mhc* sequence [GenBank accession number AF042349.1] and degenerate primers, which were adapted after [48], were used. Primer sequences are listed in the Additional file 1. PCR fragments were gel purified, subcloned into pGEM-T Easy vectors (Promega, Milan, Italy), and transformed using One Shot Top10 chemically competent cells (Invitrogen Life Technologies, Milan, Italy). Sanger sequencing was performed by a genetic analyzer 3130 (Applied Biosystems Life Technologies, Milan, Italy), and sequences were identified using BLASTx alignment program of the NCBI.

### Gene sequence alignment and orthology analysis

Amino acid sequence data of bilaterian *Myosin* gene orthologues were retrieved from NCBI and aligned in MacVector v11.0 (MacVector, Inc., Cary, NC). Aligned sequences were manually corrected for errors and Bayesian analysis was performed in MrBayes v3.1.2 [49] to infer orthology assignments. Analysis was executed using mixed models, 4 independent runs with 4 chains sampled every 100th generation and for 500,000 generations in total. The majority consensus tree was generated with a burnin value of 250. GenBank accession numbers of bilaterian phyla used in the analysis are listed in the Additional file 2. The gene tree was visualized using the program FigTree v1.4.2 (http://tree.bio.ed.ac.uk/software/figtree/) and edited with Adobe Illustrator (Adobe Systems Incorporated, San Jose, CA, USA). An amino sequence alignment of the head and tail region of cephalopod-specific *Mhc* gene orthologues was performed using MacVector v11.0.

### Probe synthesis and whole-mount *in situ* hybridization

Digoxigenin (DIG)-labeled antisense hybridization probes were synthesized using the DIG RNA labeling kit (Roche Diagnostics Corporation, Milan, Italy) with either SP6 or T7 polymerase following the manufacturer’s protocol. Gene-specific probes were used at the following lengths and concentrations: *Ov-MHC*, 1,835 bp at 0.1 ng/μL; *Ov-Actin*, 1,006 bp at 0.1 ng/μL; *Ov-Tm*, 840 bp at 1.0 ng/μL. Whole-mount *in situ* hybridization experiments were performed as previously described by Lee et al. [50] with some modifications. Embryos were rehydrated in a graded methanol series, rinsed 5 times 5 min in PTw (1× PBS, 0.1% Tween-20), and digested in 0.01 mg/mL proteinase K (Roche Diagnostics Corporation, Milan, Italy) in PTw for 6 to 20 min at RT. After post-fixation in 4% PFA in ASW for 1 h at RT and 5 washes for 5 min in PTw at RT each, embryos were heated to 80°C in PTw to inactivate endogenous alkaline phosphatase. Specimen were washed with hybridization buffer (50% formamide; 5× saline-sodium citrate (SSC), pH 4.5; 10% Denhardt’s solution; 1% SDS; 125 μg of salmon sperm DNA; 62.5 μg of yeast RNA) and incubated at hybridization temperature (65°C) for at least 4 h or overnight. Hybridization with the DIG-labeled *in situ* probes was performed at hybridization temperature over night. In order to test for nonspecific labeling, negative control experiments were performed for each condition using hybridization buffer only without probe. After hybridization, embryos were washed for 20 min in hybridization buffer; for 15 min each in 75% hybridization buffer and 25% 2× SSC, 50% hybridization buffer and 50% 2× SSC, 25% hybridization buffer and 75% 2× SSC, 100% 2× SSC; and twice for 30 min in 0.05× SSC at hybridization temperature. Embryos were then rinsed for 10 min each at RT in 75% 0.05× SSC and 25% PTw, 50% 0.05× SSC and 50% PTw, 25% 0.05× SSC and 75% PTw, and 100% PTw. Subsequently, embryos were washed 5 times for 5 min each in PBT (1× PBS, 0.1% BSA, 0.2% Triton X-100) at room temperature and blocked in blocking buffer (10% fetal bovine serum in maleic acid buffer: 0.1 M maleic acid, 0.15 M NaCl, 0.1% Tween-20) for 2 h at room temperature. Embryos were incubated in anti-DIG-AP antibody (1:2,500; Roche Diagnostics Corporation, Milan, Italy) at 4°C overnight and washed 8 times for 10 min each in PBT at room temperature. Probes were developed by incubating embryos in detection buffer (100 mM NaCl, 100 mM Tris, pH 9.5, 3.75) with a nitro blue tetrazolium (NBT) and 5-bromo-4-chloro-3-indolyl phosphate (BCIP) solution (Roche Diagnostics Corporation, Milan, Italy).

### Microscopy

Confocal imaging was performed using a Leica SP5 inverted confocal laser microscope (Leica) and three-dimensional reconstructions were generated using ImageJ. Histology and *in situ* images were obtained using an Olympus BX51 upright microscope with an Olympus Microfire digital camera. In all experiments, either arm II or III was visualized.

## Results

### Embryonic development and appearance of the arm crown in *O. vulgaris*

The embryonic development of *O. vulgaris* including the general arm crown formation was first described by Naef [42] and will be briefly summarized in this paragraph. Like all cephalopods, the *O. vulgaris* embryo exhibits a direct development, which takes about 35 days at 20°C. Embryos develop by bilateral, meroblastic cleavage in which the first cleavage furrow sets the primary body axis (Figure 1C, stage 2). During epibolic gastrulation, the yolk gets covered by a thin sheet of cells (the outer yolk sac) while the embryo proper is forming at the animal pole of the egg (Figure 1C, stage 11). Shortly thereafter, first organ primordia appear as epithelial thickenings, which in the process of organogenesis increase in size and complexity (Figure 1C, stage 16 - hatchling). The *O. vulgaris* hatchling is considered a paralarva, which undergoes a pelagic phase before settling to the sea floor.

The arm crown primordium appears right before the end of gastrulation as two bands of cells on each side of the egg’s ‘equator.’ Both bands are situated closer to the posterior part of the embryo, resulting in a larger gap on the anterior side (Figure 1C, stage 16). As the embryo expands over the yolk, the arm crown rudiment becomes more distinct and subdivides into four arm fields on each side of the embryo (Figure 1C, stages 18 to 19) [42].

### Embryonic formation and differentiation of the *O. vulgaris* arm crown

**Figure 2:**
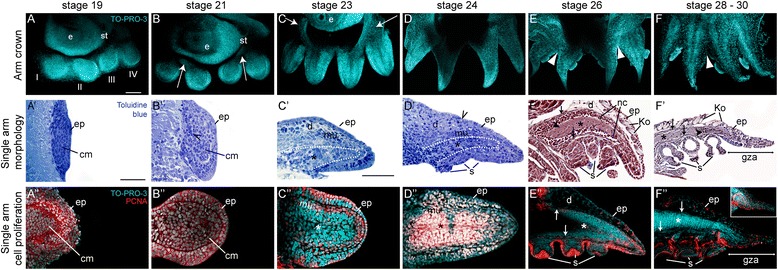
Formation of the octopus arm crown and development of the arm buds. **(A to F)** Confocal projections of arm crowns labeled with TO-PRO-3 to visualize the nuclei (cyan). Embryos are oriented in a lateral view, with anterior to the left. **(A′ to F′)** Sagittal histological sections through individual arms stained with toluidine blue **(A′ to D′)** and Masson’s trichrome stain **(E′ to F′)**. **(A″ to F″)** Merged image stacks of median aboral **(A″ to D″)**, sagittal **(E)″** and parasagittal **(F″)** sections of individual arms stained with an antibody against PCNA to visualize proliferating cell nuclei (red) and TO-PRO-3 to highlight cell nuclei (cyan). Inset in **(F″)** shows a close-up of the entire confocal stack of the arm’s tip. Individual arm buds are oriented with proximal to the left. White arrows in **(B)** to **(C)** point at tissue contributing to the eye lid; white arrowheads in **(E)** to **(F)** denote the velar webs. Black open arrowhead in **(B′)** points out the location of a cluster of darker stained, denser cells and in **(D′)** denotes the basal cells of the future Kölliker’s organ. Dotted lines are indicating the outline of the forming axial nerve cord in **(C′)** to **(D′)**; asterisk marks the general area of the axial nerve cord. Arrows are pointing at longitudinal muscle fibers; closed arrow heads are marking transverse muscle fibers in **(E′)** to **(F′)** and **(E″)** to **(F″)**. Abbreviations: cm, cell mass; d, dermis; ep, epithelium; e, eye; gza, growth zone of the arm; Ko, Kölliker’s organ; mu, muscle layer; nc, neuronal cells; s, sucker; st, statocyst. Scale bars: A (refers to **A** to **F**): 100 μm; **A**′ (refers to **A**′, **B**′ and **A**″, **B**″), **C′** (refers to **D**′ to **F**′ and **D**″ to **F**″): 50 μm.

In order to understand the basic dynamics of octopus appendage development, we analyzed the growth, formation, and differentiation of single arms within the arm crown by studying their histology and cell proliferation pattern. We thereby focused on stages right after the arm fields had formed and for continuity of the study analyzed the morphology of either arm II or arm III. The terminology used for describing the arm axes is summarized in Figure 1B. As soon as the arm fields are established, individual arm rudiments can be distinguished as bulges of moderate size at which arm rudiment III appears before rudiments II and IV and arm pair I remains developmentally delayed until stage 19 (Figure 2A). After this initial asynchronous development, all arms show a similar level of maturity during subsequent stages. Early arm rudiments consist of a few layers of undifferentiated cells enclosed by an epithelium (Figure 2A′), both of which are heavily proliferating (Figure 2A″).

In the following course of development, the arm bulges set themselves apart from the original band-like structure and can readily be distinguished by their unique position within the arm crown (Figure 2B). At this stage, the arm buds are made up by an epithelium consisting of cells similar in shape and size, encircling an underlying differentiating cell mass (Figure 2B′). Unlike the epithelium, these underlying cells are composed of several inhomogeneous cell types. In particular, we observed cell nuclei towards the proximal and central parts of the arm buds which are slightly darker, smaller, and rounder (Figure 2B′, open arrowhead). Similar types of cells in these regions were previously described as neuroblast cells migrating from the base and lateral edges of the epithelium into the arm primordium in the *O. vulgaris* embryo [44]. All cell types continue to proliferate strongly within the now hemispherical arm rudiments (Figure 2B″).

While the arm crown slowly contracts, wedge-shaped tissue starts to grow from the anterior proximal base of arm bud II and posterior proximal base of arm bud III, towards the anterior and posterior part of the eye (Figure 2C, arrows), which later envelopes the eyes to form the primary eyelid [28]. Within the arm bud, the previously seemingly undifferentiated cell mass starts to clearly organize into distinct tissue layers at stage 23 (Figure 2C′). Underneath the epithelium, we observed a loose layer of darker stained and larger cells adjacent to an area of densely packed elongated cells within the areas of the future dermis and muscle mass, respectively. A central mass of small and rounded cells is making up the future axial nerve cord. In addition to an organization into distinct cell layers, we noted a dramatic change of shape from a hemispherical arm bud to an elongated arm. Cell proliferation is more pronounced in the lateral epithelium and at the distal tip of the arm bud in an area underneath the epithelium (Figure 2C″).

As the embryo increases in complexity, the four arm pairs begin to encircle the originally anterior situated mouth (Figure 2D). At this stage, we could clearly distinguish between the epithelium, dermis, muscle layer, and axial nerve cord (Figure 2D′). Several cells have submersed under the epithelium and differentiated into the basal cells of what will become the Kölliker’s organs. These tegumentary structures are a larval feature unique to most incirrate octopods, which assist in hatching and facilitate post-hatching swimming behavior [51] (Figure 2D′, open arrowhead). Underneath the epithelium, the thick, loose dermis is taking up a large part of the proximal, aboral fraction of the arm adjacent to which the dense muscle layer becomes evident. The axial nerve cord consists mostly of round cell bodies; however, at this stage, a ganglionic structure is not visible yet. Proliferation is most pronounced in the lateral and proximal parts of the epithelium, the muscle layer, and the cell bodies within the axial nerve cord (Figure 2D″).

Over the next stages of development, the morphology of the arm crown matures considerably. The individual arms become connected by a velar web, which surrounds the proximal base of the arm (Figure 2E, arrowheads). Within the epithelium, the basal cells of the Kölliker’s organs have multiplied and formed into cup-shaped invaginations secreting spine-like setae (Figure 2E′). The dermis underneath the epithelium has developed into a loose, fibrous layer. A thin layer of longitudinal and transverse muscle fibers borders a dense layer of differentiating cells, which envelopes the axial nerve cord. These differentiating cells were previously described by Kier [52] as nerve cell bodies; however, cells within this area in the tentacle of the cuttlefish have been considered as differentiating muscle cells (myocytes) by Grimaldi et al. [53]. Given the position of these cells within the arm, we believe that they may constitute a combination of both. Proliferation is now mostly restricted to the distal tip of the arm, to the newly forming longitudinal muscle layer and the suckers (Figure 2E″).

At hatching stage, the mouth comes to lie in the center of the four pairs of seemingly homonomous arms (data not shown) while the velar webs further increase in complexity (Figure 2F, arrowhead). The aboral side of the arm is entirely covered in Kölliker’s organs, which start to break through the epithelium. Underneath the dermis, the layers of longitudinal muscle fibers have increased and transverse muscle fibers are more abundant. The amount of cells surrounding the axial nerve cord as well as those within the neuropil is reduced. Furthermore, the axial nerve cord has formed into three ganglionic regions, corresponding to the three suckers on the oral side of the arm. In terms of overall shape, we noticed that the distal end of the arm becomes drawn-out into a pointed tip, typical for adult animals. This area was termed the growth zone of the arm (gza) or flagellum by Naef [42]. Cell proliferation is mostly restricted to this area, to the muscle layer right beneath the dermis and the suckers (Figure 2F′). Interestingly, the overall complexity decreases from the proximal base towards the distal tip of the arm with the tip remaining in a state of development comparable to stage 26 (Additional file 3).

### Arm bud outgrowth and elongation

**Figure 3:**
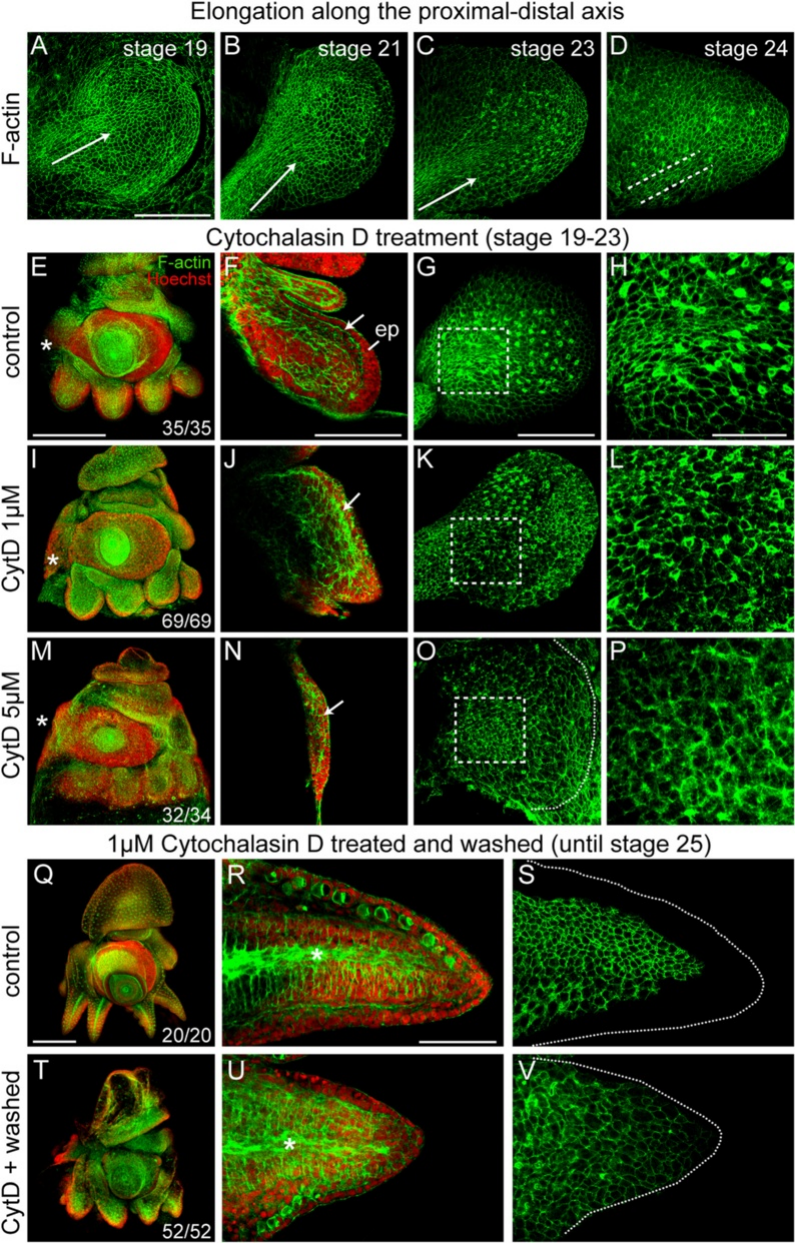
Elongation of the arm. **(A to D)** Confocal projections of surface epithelium stained with phallacidin to visualize F-actin, aboral view, proximal to left. Arrows and dotted lines **(D)** point into the direction of cell elongation. **(E)** to **(H)** arm crown **(E)** and close-up **(F)** to **(H)** of control embryo. **(I**) to **(L)** and **(M)** to **(P)** embryos treated with 1 or 5 μM CytD. **(E, I, M)** embryos stage 23 stained with phallacidin (green) and Hoechst to visualize nuclei (red), lateral view, anterior to the left. Asterisks indicate position of mouth. **(F, J, N)** Sagittal confocal sections of arm buds corresponding to embryos in **(E, I, M)**, proximal to left. Arrows point at basement membrane. **(G, K, L)** Confocal stacks of arm buds in **(F, J, N)** showing the surface epithelium, oral view. Dotted line in **(O)** indicates outer margin of arm bud. Boxed regions are magnified in **(H, L, P)**. Row **(Q)** to **(S)** control embryo **(Q)** and arm at stage 25 **(R to S)**. Row **(T)** to **(V)** embryo treated with 1 μM CytD until stage 23, washed and left to develop until stage 25 **(T)** and arm at stage 25 **(U to V)**. **(Q, T)** embryos stage 25 stained with phallacidin (green) and Hoechst (red), lateral view, anterior to the left. **(R, U)** Confocal stacks of arm buds in **(Q, T)** and surface epithelium of the same arms stained with phallacidin **(S, V)**, oral median view. Numbers in the right lower corner in **(I, M, T)** shows number of embryos with this phenotype in relation to number of embryos treated. Abbreviations: e, eye; ep, epithelium. Scale bars: E (refers to **E, I M**), Q (refers to **Q, T**): 300 μm; A (refers to **A to D**), F (refers to **F, J, N**), G (refers to **G, K, O**), H (refers to **H, L, P**), R (refers to **R, S** and **U, V**): 100 μm.

During the early stages of arm development (stages 19 to 23), we observed a drastic elongation of the spherical arm bulge along its proximal-distal axis. Since axes elongations are often accompanied by epithelial cell dynamics in embryonic development [54-58], we monitored the shape and orientation of cells stained for F-actin during early arm formation. Rows of elongated, epithelial cells appear at the proximal base of the arms and are extending obliquely into the arm buds from stage 19 until stage 23 (Figure 3A,B,C; Additional files 4, 5, and 6). At stage 24 (Figure 3D; Additional file 7), only remnants of these elongated cells remain (dashed lines).

To understand whether an elongation of epithelial cells can indeed contribute to the elongation process of the early arm buds, we disrupted actin polymerization within the cytoskeleton using Cyt D. We performed treatments with two concentrations of Cyt D (1 or 5 μM) for the duration of early arm outgrowth and elongation (stages 19 to 23). Embryos raised in control medium form anatomically normal and elongated arms (*n* = 35, 100%; Figure 3E). The basement membrane distinctly separates the epithelium from the underlying cell mass (Figure 3F, arrow) and the surface epithelium includes elongated cells (Figure 3G,H). In contrast, the arms of embryos treated with 1 μM Cyt D fail to fully elongate and appear malformed (*n* = 69, 100%; Figure 3I). In particular, the arms’ lengths, widths, and thicknesses are reduced (*n* = 7; ANOVA, *P* < 0.05; Additional file 8A) and the basement membranes are highly disorganized (Figure 3J). However, the average cell number in these arms does not significantly differ from the cell number in the control arms (*n* = 6; ANOVA, *P* = 0.164; Additional file 8B). Furthermore, epithelial cells on the oral surface of the arms are not elongated (Figure 3K,L). Cell shape measurements show a clear reduction in the length to width ratio with a decrease in length of otherwise elongated epithelial cells (*n* = 40; ANOVA, *P* < 0.05; Additional file 8C). Arm buds of embryos treated with 5 μM Cyt D fail to grow out and remain in a developmental state comparable to stage 19 embryos (*n* = 34, 94%; Figure 3M). The arm bulges consist of a thin epithelium separated by a discontinuous basement membrane from the few underlying cell layers (Figure 3N). Arm length, width, and thickness in these arms is significantly reduced when compared to control embryos (*n* = 14; ANOVA, *P* < 0.05; Additional file 8A) as well as the number of cells (*n* = 7; ANOVA, *P* < 0.05; Additional file 8B). Cells on the surface epithelium are not elongated but round and do not show clear cell boundaries (Figure 3O,P). Cell shape measurements show a clear reduction in the length to width ratio with a decrease in length in these embryos (*n* = 40; ANOVA, *P* < 0.05; Additional file 8C).

To test whether the observed epithelial cell changes during this particular developmental window (stages 19 to 23) are a crucial factor for the elongation of the arm, Cyt D-treated embryos were washed in normal sea water and allowed to develop until control embryos were at stage 25 (Figure 3Q,R,S). Embryos initially treated with 5 μM Cyt D do not survive past stage 24 (*n* = 33, 100%). Embryos initially treated with 1 μM Cyt D remain shorter in overall size (*n* = 52, 100%; Figure 3T). Furthermore, even though the arms continue to differentiate, they fail to elongate (Figure 3U). Measurements of the arms’ dimensions in the treated *versus* control group confirm this observation showing a tendency of the treated arms to be shorter but thicker (*n* = 11; *t* test, *P* < 0.05; Additional file 8D). The surface epithelium at this stage does not include elongated cells in the control embryos (Figure 3S) and elongated cells are equally absent from the surface epithelium of the initially treated embryos (Figure 3V). Altogether, these results suggest that actin polymerization is crucial for the elongation of epithelial cells and the concomitant elongation of the embryonic arm during a specific period of arm formation.

### Formation of the embryonic arm musculature

The *O. vulgaris* adult arm consists of a complex arrangement of muscle layers (Figure 1A), which are composed of large, mononucleated muscle cells with oblique striation (Additional file 9A). To understand how this network is established during embryonic development, we examined the first appearance of muscle cells, formation of muscle fibers, and their subsequent arrangement into distinct muscle layers within the developing arm buds.

**Figure 4:**
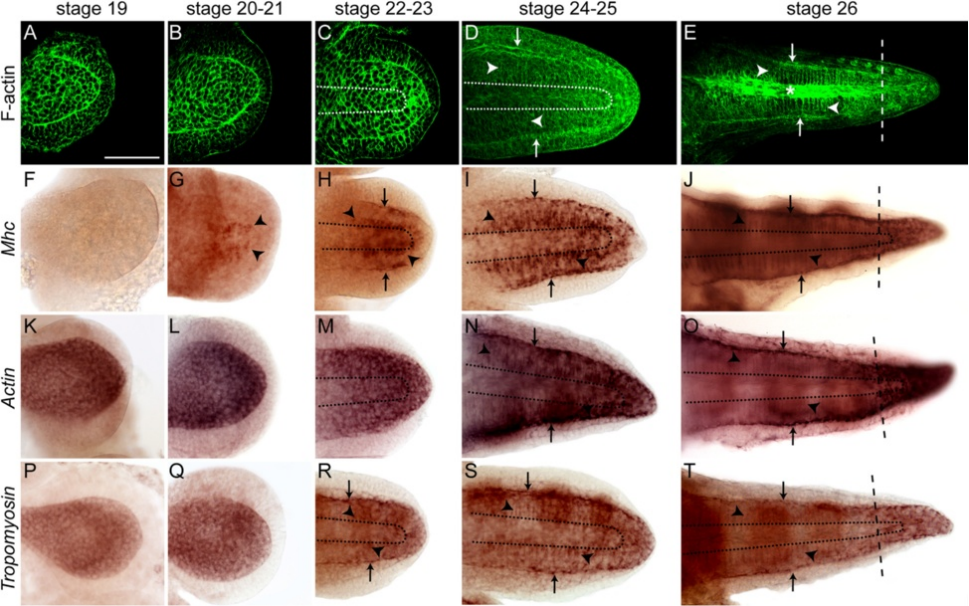
Development of the arm’s musculature. Developmental stages of the arms shown in each column are indicated at the top margin, staining/gene name of gene expression (purple) shown in each row is indicated on the left margin. Arms are displayed in an oral view with proximal to the left. **(A to E)** First muscle fibers detected by phallacidin staining appear at stages 24 to 25 **(B)** in the area of the longitudinal (arrows) and transverse muscle layer (arrowheads). At stage 26 **(E)**, both muscle layers increase in complexity. **(F to J)** *Ov-Mhc* is not detected in early limb bulges **(F)** and first appears at stages 20 to 21 in large cells (arrowheads) within the area of the future transverse muscle layer **(G)**. From stages 22 to 23 onwards, *Ov-Mhc* is also expressed in the area of the future longitudinal muscle layer (arrows) **(H to J)**. **(K to O)** *Ov-Actin* is expressed throughout the arm’s development. From stages 24 to 25, it strongly localizes in the areas of the future transverse (arrowheads) and longitudinal muscle layers (arrows) **(N to O)**. **(P to T)** *Ov-Tm* is expressed in the inner cell mass during early arm outgrowth **(P, Q)**. Starting stages 22 to 23, it becomes localized to cells in the future longitudinal (arrows) and transverse muscle layers (arrowheads) **(R to T)**. Asterisk in **(E)** and dotted line indicate the area of the axial nerve cord; dashed line marks the area of the growth zone of the arm. Arrowheads denote muscle cells/muscle fibers in the area of the transverse muscle layer; arrows point at muscle cells/muscle fibers in the area of the longitudinal muscle layer. Scale bar (refers to **A** to **T**): 100 μm.

We first analyzed the morphology of *O. vulgaris* embryonic muscle cells in cell culture so as to readily identify these cells in the context of the developing tissue. We then followed the appearance and subsequent organization of embryonic muscle cells into different muscle layers by staining embryonic arms for F-actin (Figure 4A,B,C,D,E). Embryonic muscle cells from late developmental stages are significantly smaller when compared to adult muscle cells and the striation is not apparent yet (Additional file 9B). We first observed these cells at stage 24, right after the arms had elongated. At this stage, muscle cells are located adjacent to the epithelium in the area of the future longitudinal muscle fibers and in a deeper tissue layer in the area of the transverse muscle fibers (Figure 4D, arrows and arrowheads) surrounding the future axial nerve cord (dotted line). Both muscle layers become more prominent at stage 26 (Figure 4E). Interestingly, muscle fibers in the distal tip within the growth zone of the arm are not organized into distinct layers (Figure 4E, dashed line). Right before hatching, the arm’s musculature consists of a thin longitudinal and an intertwined, sparse transverse muscle layer (Additional file 9C).

### Muscle-specific genes and phylogenetic analysis

During invertebrate and vertebrate muscle development, muscle precursor cells (myoblast cells) determined to differentiate into mature muscle cells (myocytes) commonly initiate the expression of muscle genes including *Myosin heavy chain*, *Actin*, and *Tropomyosin* [59-63]. These muscle genes are often activated at different times of muscle formation, dependent on muscle fiber type and location. For instance, in the gastropod *Haliotis rufescens*, *Tropomyosin* mRNA accumulates up to 7 days prior to myofibril assembly [64]. In order to examine where early myocytes originate from and how they differentiate into mature myofibers, we therefore cloned and studied the gene expression patterns of the well-conserved muscle genes *Myosin heavy chain*, *Actin*, and *Tropomyosin.*

Full-length gene orthologs of *O. vulgaris Actin* (*Ov-Actin*) and *Ov-Tm* were previously characterized by Ochiai et al. [65] and Motoyama et al. [66], respectively. Both *Ov-Actin* and *Ov-Tm* were isolated from the *O. vulgaris* arm musculature. While the deduced *Ov-Actin* amino acid sequence shows strong sequence identity to other invertebrate and vertebrate actins, *Ov-Tm* is highly cephalopod specific. We further identified two fragments of the *Ov-Mhc* gene, the deduced amino acid sequence of which shows strong sequence similarities to the head and a tail region of known cephalopod *Myosin heavy chain* gene orthologues (Additional file 10). A maximum likelihood (BS) analysis supports a specific assignment of the *Ov-Mhc* gene to a distinct clade of MyosinII subfamily orthologs from representatives of cnidaria, lophotrochozoa, ecdysozoa, and chordata with high posterior probabilities (Additional file 11).

### Whole-mount *in situ* expression patterns

*Ov-Mhc* expression is first detected in large, spindle-shaped cells (early myocytes) at stages 20 to 21 in the area of the future transverse muscle layer (Figure 4G, arrowheads; Additional file 9D). At stages 22 to 23, the number of cells expressing *Ov-Mhc* in this region surrounding the future axial nerve cord increases (Figure 4H dotted line, arrowheads) and expression also appears in fewer cells adjacent to the epithelium in the area of the future longitudinal muscle layer (Figure 4, arrows). The *Ov-Mhc*-positive cells at this stage include cells, which form fibrous extensions (late myocytes). At stages 24 to 25, the *Ov-Mhc* expression intensifies in both regions and is visible in embryonic muscle fibers (Figure 4I, arrowheads and arrows; Additional file 9E) as well as early myocytes, which form at the tip. At stage 26, we detected *Ov-Mhc* expression in both the longitudinal and transverse muscle layer, respectively (Figure 4J, arrowheads and arrows; Additional file 9 F) as well as newly forming muscle cells in the growth zone of the arm (Figure 4J, dashed line; Additional file 9G).

We observed strong *Ov-Actin* expression within the entire inner cell mass of the early arm bulge and elongating arm bud (stages 19 to 23, Figure 4K,L,M). At stages 24 to 25, the expression becomes clearly localized to the muscle fibers of the transverse and longitudinal muscle layers (Figure 4N, arrowheads and arrows). This expression becomes even more pronounced at stage 26 and is mostly localized to maturing muscle layers at this stage (Figure 4O, arrowheads and arrows). We observed a less localized expression in the growth zone of the arm (Figure 4N,O, dashed lines).

At stages 19 to 21, we detected *Ov-Tm* expression in the cell mass underneath the epithelium (Figure 4P,Q). At stages 22 to 23, a weak *Ov-Tm* expression becomes localized to the forming longitudinal and transverse muscle fibers (Figure 4R, arrowheads and arrows). The expression intensifies in the following developmental stages and is mostly detectable within the layers of the differentiating longitudinal and transverse muscle layers (Figure 4S,T, arrowheads and arrows). *Ov-Tm* expression within the growth zone of the arm remains less localized (4S,T, dashed line).

### Formation of the sucker

**Figure 5:**
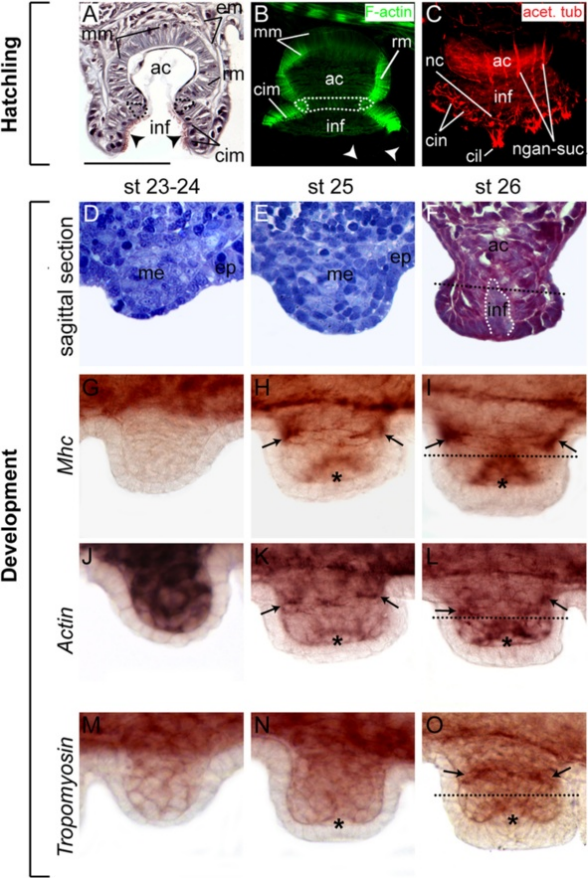
Embryonic formation of the sucker. **(A to C)** Morphology of the sucker at hatching stage. **(A)** Sagittal histological section through oral part of arm stained with Masson’s trichrome stain. **(B, C)** Confocal projection of sucker in a sagittal position stained with phallacidin to visualize F-actin **(B)** and of the whole sucker stained with an antibody against acetylated tubulin to visualize nerve tracts **(C)**. **(D to O)** Developmental stages for the suckers shown in each column are indicated at the top margin, staining/gene name of gene expression (purple) shown in each row is indicated on the left margin. **(D to F)** Sagittal histological sections through the oral part of the arms stained with toluidine blue **(D, E)** and Masson’s trichrome stain **(F)**. **(G to I)** Ov-Mhc **(J to L)** Ov-Actin and **(M to O)** Ov-Tm expression during sucker development. Dotted lines in **(A, B)** encircle the position of the sphincter muscle. Dotted white line in **(F)** highlights the outline of the invaginated acetabulum and infundibulum. Dotted black line marks the border between acetabulum and infundibulum. Arrow points at extrinsic circular muscle. asterisk denotes the forming intrinsic sucker muscle. Abbreviations: ac, acetabulum; cil, cilia; cim, circular muscle; cin; circular infundibulum nerve; em, extrinsic musculature; ep, epithelium; inf, infundibulum; me, mesoderm; mm, meridional muscle; ngan-suc, nerves connecting ganglion and sucker; rm, radial muscle. Scale bar in A (refers to **A to O**): 20 μm.

The octopus sucker is an important manipulative and chemosensory structure on the oral side of the arm, allowing the animal to probe its environment and perform fine motor tasks. At hatching stage, the sucker is made up of radial, meridional, and circular muscle fibers and a rudimentary sphincter muscle, surrounded by the extrinsic musculature, which connects the sucker to the arm proper (Figure 5A,B). The nervous system of the sucker at this stage is almost completely formed and consists of a nerve ring surrounding the sucker rim and bundles of nerves connecting the sucker with the sucker ganglion and the nervous system of the arm (Figure 5C).

In order to understand how the musculature of this complex structure is formed, we examined the expression patterns of the aforementioned muscle-specific genes during the embryonic formation of the suckers. Octopus sucker development was previously described by Fioroni [67] and Nolte and Fioroni [68] and is summarized in Figure 5D,E,F. First sucker rudiments appear at stage 23 as small epithelial outgrowths, which include both a mesodermal and an ectodermal component (Figure 5D). These outgrowths soon increase in size (Figure 5E) and develop a primordial acetabulum, which invaginates from the epithelium surrounding the sucker rudiment (Figure 5F, white dotted line).

During sucker development, *Ov-Mhc* and *Ov-Actin* are first expressed at stage 25 within the extrinsic acetabulo-brachial musculature (Figure 5G,H,J,K, arrows). Both genes are also expressed in maturing muscle cells of the future acetabulum, which is just about to invaginate (Figure 5H,K, asterisk). At stage 26, all three muscle-specific genes examined (Figure 5G,H,I,J,K,L,M,N,O) are strongly expressed within the extrinsic circular musculature (Figure 5I,L,O, arrows) and the circular muscle of the now invaginating acetabulum (Figure 5I,L,O, asterisk).

## Discussion

**Figure 6:**
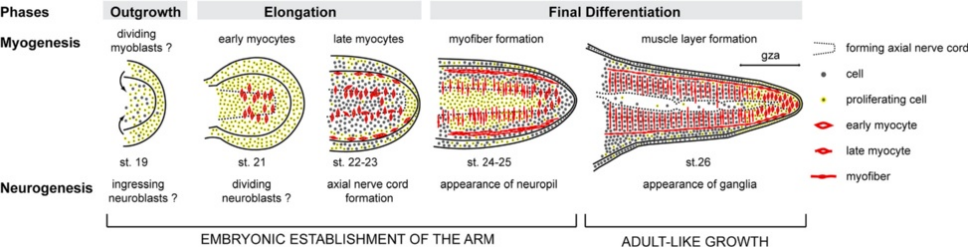
Schematic summary of the main events during the embryonic formation of the octopus arm. Arms are illustrated in an oral view with proximal to the left. Octopus arm formation can be divided into distinct developmental phases, each corresponding to specific developmental events. Early embryonic development establishes the arm, which switches to an adult type of growth once the main tissue types have differentiated. Abbreviations: gza, growth zone of the arm.

In this study, we presented a first, detailed description of the embryonic formation and differentiation of the *O. vulgaris* appendages and thereby established a basis for further studies on appendage patterning, growth, and differentiation in a representative of the lophotrochozoan super phylum. The octopus arm formation is a dynamic process, which can be subdivided into distinct phases, each one corresponding to a specific developmental event. These phases include an initial outgrowth of individual arm bulges from epithelial thickenings, an elongation along their proximal-distal axes, and a final phase of differentiation (Figure 6).

### Early outgrowth and elongation of the octopus arm

A prevalent mode of appendage formation in animal models studied so far is the formation of mesodermal and epithelial thickenings, rapid proliferation, and a subsequent elongation along the proximal-distal axis [5,55,69]. This process is commonly accompanied by mechanisms including oriented cell shape changes, oriented cell division, convergent extension, and cell motility [54-57,70-74]. For instance, the tentacles of the sea anemone *Nematostella vectensis* originate from an epithelial thickening on the oral surface of the embryo, the elongation of which is achieved by ectodermal thinning and the linear arrangement of epithelial cells along the proximal-distal axis [55]. Furthermore, the adult leg of the fruitfly *D. melanogaster* elongates from the imaginal leg disc with the help of apical and basal cell shape changes from elongate to spherical [5,54], while the formation of the vertebrate limb bud seems to be mediated by the directional migration of mesenchymal cells into the prospective limb field and their oriented cell division [72,75]. Here, we show that dynamic cellular processes are likewise involved in the initiation, growth, and elongation of the octopus arm primordia. The octopus arm crown first appears as an epithelial thickening from which individual arm bulges grow out. The subsequent elongation of the originally spherical arm primordium coincides with an arrangement of elongated cells in a slightly oblique angle along the proximal-distal axis. Our data suggests that actin is required for the elongation of these cells, which in turn is crucial for the elongation of the entire arm. Since disruption of actin polymerization also impacts cell division, directed cell proliferation might equally play a role in the elongation process of the arm, which would need to be investigated further. Most interestingly, elongation during early arm formation (stages 19 to 23) seems to be significant and cannot be fully compensated for later on. A similar observation was made by Gros et al. [76] who show that the initial elongation from an early arm bulge to a pedal-shaped limb bud happens in a relatively short time window during vertebrate early limb formation. Taken together, we suggest that epithelial cell dynamics and cellular behaviors might indeed reflect a common feature of appendage formation throughout the animal kingdom, as suggested by Fritz et al. [55]. Wnt, Fgf, and Notch signaling have been indicated as important regulators of appendage outgrowth and elongation in various animal phyla [17,55,76-80]. Comparative studies on these gene regulatory networks might, therefore, open up new perspectives on the evolution of animal appendages.

### Differentiation of the tissue layers

During the arm’s differentiation, the main tissue types are laid out; however, their maturing is delayed until after hatching. This appears to be due to the fact that *O. vulgaris* hatchlings undergo a pelagic phase during which their arms elongate extensively and both nervous and muscular elements reach adult-like maturity only when settling to their benthic lifestyle [41,81,82].

In particular, during the arm’s early growth phase, we observed cell clusters in the lateral and proximal portion of the arm bud, which according to Marquis [44] most likely constitute ingressing neuronal precursor cells. In the sepiid cephalopod *Sepia officinalis*, cells of the future axial nerve cord within this central region express the cuttlefish homolog of the early neuron-specific RNA binding protein ELAV, *Sof-elav1*, shortly thereafter [83]. It is, therefore, possible that this cell cluster indeed constitutes neuronal precursor cells, which originate from the epithelium, migrate in and initiate differentiation as soon as they have reached their final position. Through rapid cell proliferation, the early axial nerve cord quickly increases in size and subsequently develops a central neuropil surrounded by maturing neurons. Even though three suckers are present at the time of hatching, the formation of the ganglionic structure of the nerve cord is only initiated (Figure 6).

Similar to the formation of the axial nerve cord, muscle formation begins relatively early during the octopus arm development. Here, we studied the expression patterns of the muscle-specific genes *Ov-Mhc*, *Ov-Actin*, and *Ov-Tm* to identify early and differentiating myocytes in the developing octopus arm. Our observations show that *Ov-Mhc* is the first of these three genes to be expressed within early spindle-shaped myocytes, long before mature myofibers are detected. Even though the longitudinal muscle layer is the more dominant muscle type at hatching stage, early myocytes first appear in the area of the future transverse muscle layer surrounding the developing axial nerve cord. Towards the end of the elongation phase of the arm, few *Ov-Mhc*-positive cells also appear in the area of the future longitudinal muscle layer. The onset of *Ov-Actin* and *Ov-Tm* localization to myocytes during subsequent stages coincides with the maturing of the early myocytes into late myocytes and subsequently into myofibers forming muscle layers during late stage development. In the *S. officinalis*’ retractile tentacle, a pool of myoblast cells expressing vertebrate-type myoblast-specific Myf5 and MyoD proteins are surrounding the axial nerve cord during late stage development (stages 26 to 30). These cells migrate centrifugally (that is, from the center towards the periphery) where they differentiate into myocytes [84]. Since vertebrate-type Myf5 or MyoD proteins are not expressed during octopus development (data not shown) and we could not identify myoblast-specific genes using degenerate PCR, we were unable to localize the origin of muscle precursor cells. Given that early myocytes first appear in an area around the future axial nerve cord and slightly later within the periphery of the arm, it is indeed possible that dividing myoblast cells surround the primordial axial nerve cord and migrate into the periphery, where they initiate differentiation. A similar situation can be observed during vertebrate limb myogenesis, where ingressed myoblast cells proliferate within the limb primordium, divide into distinct cell masses, and differentiate into mature myocytes (reviewed in [85-87]). However, in contrast to *S. officinalis*, the events observed during octopus arm muscle development happen rather early during arm outgrowth, while at stage 26, most early myocytes are localized to the proliferating growth zone of the arm. Furthermore, the octopus arm and the cuttlefish tentacle are anatomically diverse structures which are composed of different sets of muscle types [32,52,88] and, therefore, possibly inept to compare. The identification of species-specific muscle precursor and myoblast markers and their expression studies will help elucidate the origin of muscle precursor cells and their determination to the myogenic lineage.

Shortly before hatching, the longitudinal muscle layer constitutes the most developed part of the arm’s musculature. This muscle layer is known to be involved in shortening and bending motions of the *O. vulgaris* arm [32], both of which are important movements for prey grasping. However, similar to the axial nerve cord, the majority of the musculature, including the rudimentary transverse and oblique muscle layers, will further differentiate during post-hatching stages [89].

### Embryonic *versus* adult-like growth

The major events of arm formation and differentiation are summarized in Figure 6. During the first phase of arm outgrowth, we observed that cell proliferation is not spatially localized but uniform. The inner cell mass of the arm at this stage presumably consists of undifferentiated neuroblast and myoblast cells. During the early phase of arm elongation, differentiation of neuronal cells and myocytes is initiated, which clearly organize into distinct regions, while still proliferating heavily. Towards the end of arm elongation and early differentiation, cell proliferation becomes more regionalized and corresponds to the area of current tissue differentiation (that is, muscle layers and axial nerve cord). During late differentiation stages, we observed a very distinct distal regionalization of proliferation within the growth zone of the arm. Maturing myocytes are mostly localized to this area. As opposed to an allometric appendage growth found in insects [90,91], surprisingly similar cell proliferation patterns have been observed during vertebrate limb formation. In mouse and chicken embryos, cell proliferation is initially uniform in early limb bulges, becomes spatially localized to myogenic areas at the onset of chondrogenesis and limb elongation, and is mostly restricted to the distal tip of the limb at later stages [92]. These parallels in proliferation patterns might reflect general requirements for the formation of a complex appendage. Specifically, after initial growth and early patterning of the limb, cell division and cell differentiation has to be kept in balance and thus becomes more localized. As soon as the appendage is established and organized into differentiating tissue layers, further development is focused on a progressive end-differentiation during late embryonic development. In octopus, the localization of cell proliferation to the distal tip during late developmental stages represents an adult-like growth type, which is maintained throughout the animal’s life time. Furthermore, the aforementioned end-differentiation of tissue layers, including the ganglionic structure and the majority of the arm’s musculature, is postponed to post-hatching stages and correlated with emerging behavioral modifications (for example, adult-like predatory arm use). A comparable dynamic was observed during the development of the squid *Sepioteuthis lessoniana*, in which the tentacle musculature and corresponding prey capture behavior only became functional 4 weeks after hatching [52].

### Development and regeneration

Interestingly, some of the features of the arm’s embryonic tissue formation seem to correspond to the tissue reorganization during regeneration. Similarities include a shift from an early isotropic, mesenchymal cell proliferation to a distally regionalized cell division pattern [40], as well as the formation of suckers as a single row of rounded papillae [38]. Therefore, the *O. vulgaris* arm offers an uncommon opportunity to comparatively study the morphological and genetic basis of appendage formation and differentiation during embryonic development, post-hatching and adult development, and adult regeneration within a single organism. Its versatility raises many questions on how this structure is patterned and whether similar pathways are utilized in the octopus arm throughout the animal’s lifetime or life stage-specific regulation is responsible for continuous growth and (re)formation. In addition, due to its position as a highly evolved mollusk within the lophotrochozoan super phylum, the octopus constitutes a perfect study organism to gain insight into the evolution of body plan innovations.

## Conclusions

The formation of octopus arms is a dynamic process, which shows similarities to the appendage formation of known model species as well as species-specific characteristics. In particular, development is divided into distinct phases, including an outgrowth of an early arm bulge by uniform cell proliferation and a subsequent elongation along the proximal-distal axis. Both events are prevalent features of appendage formation throughout the animal kingdom and commonly include actin-mediated cell dynamics. Interestingly, in octopus, these cell dynamics seem to be confined to a specific developmental time window during early arm formation (stages 19 to 23) and cannot be fully compensated for later on. Arm elongation marks the onset of early cell differentiation during which cell proliferation becomes regionalized and corresponds to the area of tissue differentiation. Once the embryonic arm is established and the general tissue framework is laid out, the arm switches to an adult-like distal growth pattern with a progressive mode of development. During this late differentiation stage, all tissue layers are initiated but final maturing and functionality is delayed to post-hatching stages. This species-specific differentiation mode parallels the octopus’ paralarval post-hatching phase. Given that the adult octopus arm is capable of regeneration, it provides a valuable model system for studying embryonic pattern formation and tissue (re)organization in a representative of the lophotrochozoa.

## Additional files

Additional file 1:
**List of specific and degenerate primers.** Primers used to clone the gene fragments of *Ov-Mhc*, *Ov-Tm*, *Ov-Actin*, and lengths of obtained sequences. Additional file 2:
**GenBank accession numbers.** List of phyla and GenBank accession numbers used in phylogenetic analysis. Additional file 3:
**Complexity of an octopus arm at hatching stage along its proximal-distal axis.** Schematic drawing of an arm at hatching stage, which illustrates the position of the histological cross sections shown in (i, ii, iii) stained with Masson’s trichrome stain. Asterisk marks the axial nerve cord. Scale bar: 20 μm. Additional file 4:
**Elongation of the arm bud.** Confocal stack through an arm bud stage 19 stained for F-actin. Additional file 5:
**Elongation of the arm bud.** Confocal stack through an arm bud stage 21 stained for F-actin. Additional file 6:
**Elongation of the arm bud.** Confocal stack through an arm bud stage 23 stained for F-actin. Additional file 7:
**Elongation of the arm bud.** Confocal stack through an arm bud stage 24 stained for F-actin. Additional file 8:
**Quantification of the morphological modifications in the arms and cells of the CytD 1 and CytD 5 treated embryos.** (A) Arm length, width, and thickness of stage 23 CytD1- and CytD5-treated embryos (*n* = 27; ANOVA, *P* < 0.05). (B) Cell number of stage 23 CytD1- and CytD5-treated embryos (*n* = 10; ANOVA, *P* = 0.164 (CytD1), *P* < 0.05 (CytD5)). (C) Cell dimension of stage 23 CytD1- and CytD5-treated embryos (*n* = 60; ANOVA, *P* < 0.05). (D) Arm length, width, and thickness of stage 25 CytD1-treated embryos (*n* = 11; *t* test, *P* < 0.05). Additional file 9:
***Octopus*** **muscle cells.** Dissociated longitudinal muscle fiber of an adult animal (A) and dissociated muscle fiber of a hatchling’s arm (B) stained with Phallacidin to label F-actin (green) and Hoechst to visualize the nuclei. Insets on the upper right show a magnification of the boxed regions. Compared to the adult muscle cell, the striation in the embryonic muscle cell is not apparent yet. (C) Merged image stack of sagittal sections of individual arm stages 28 to 30 stained with phallacidin to visualize F-actin. Arrows point at longitudinal muscle layer, arrowheads denote the transverse muscle layer. Asterisk marks the area of the axial nerve cord. Dashed line indicates the area of the growth zone of the arm. (D) Close-up of stages 20 to 21 *Ov-Mhc in situ* hybridization. Dotted line encircles the area of neuronal precursor cells of the future axial nerve cord. (E) Close-up of stages 24 to 25 *Ov-Mhc in situ* hybridization. Dotted lines encircle the axial nerve cord. (F) Close-up of stage 26 *Ov-Mhc in situ* hybridization. Dashed line confines an area of neuronal and overlying transverse muscle cells adjacent to the axial nerve cord. (G) Close-up of the growth zone of a stage 26 arm. Abbreviation: gza, growth zone of the arm. Scale bars: A, C, D (refers to D to G): 100 μm, B: 50 μm. Additional file 10:
**Sequence alignment of the deduced** ***Ov-Mhc*** **amino acid sequences with known cephalopod orthologs.** Abbreviations and accession numbers: Lpe, *Loligo pealeii* **(**AAC24207.1); Obi, *Octopus bimaculoides* (CDG41623.1); Ovu, *Octopus vulgaris*; Sof, *Sepia officinalis* (CDG41619.1). Additional file 11:
**Phylogenetic tree of the Myosin superfamily.** *Ov-Mhc* is specifically assigned to a distinct clade of MyosinII subfamily orthologs from representatives of cnidaria, lophotrochozoa, ecdysozoa, and chordata. Numbers represent the posterior probabilities.

## Abbreviations

ac: acetabulum
ar: artery
ch: chromatophore
cil: cilia
cim: circular muscle
cin: circular infundibulum nerve
cm: cell mass
d: dermis
e: eye
em: extrinsic musculature
ep: epithelium
f: funnel
gza: growth zone of the arm
in: intramuscular nerve
inf: infundibulum
Ko: Kölliker’s organ
m: mantel
me: mesoderm
mm: meridional muscle
mu: muscle layer
nc: neuronal cells
ngan-suc: nerves connecting ganglion and sucker
rm: radial muscle s, sucker
st: statocyst
tr: trabeculae
y: yolk
yp: yolk plug

## References

[1] Schneider I, Shubin NH (2013). The origin of the tetrapod limb: from expeditions to enhancers. Trends Genet.

[2] Shubin N, Tabin C, Carroll S (1997). Fossils, genes and the evolution of animal limbs. Nature.

[3] Shubin N, Tabin C, Carroll S (2009). Deep homology and the origins of evolutionary novelty. Nature.

[4] Tabin CJ, Carroll S, Panganiban G (1999). Out on a limb: parallels in vertebrate and invertebrate limb patterning and the origin of appendages. Am Zool.

[5] Kalm L, Fristrom D, Fristrom J. The making of a fly leg: a model for epithelial morphogenesis. Bioessays. 1995;17(8):693–702. 10.1002/bies.950170806 7661850

[6] Morata G (2001). How *drosophila* appendages develop. Nat Rev Mol Cell Biol.

[7] Minelli A (2002). Homology, limbs, and genitalia. Evol Dev.

[8] Niswander L (2003). Pattern formation: old models out on a limb. Nat Rev Genet.

[9] Fernandez-Teran M, Ros MA (2008). The apical ectodermal ridge: morphological aspects and signaling pathways. Int J Dev Biol.

[10] Zeller R, Lopez-Rios J, Zuniga A (2009). Vertebrate limb bud development: moving towards integrative analysis of organogenesis. Nat Rev Genet.

[11] Towers M, Tickle C (2009). Growing models of vertebrate limb development. Development.

[12] Wootton RJ (1999). Invertebrate paraxial locomotory appendages: design, deformation and control. J Exp Biol.

[13] Jockusch EL, Williams TA, Nagy LM (2004). The evolution of patterning of serially homologous appendages in insects. Dev Genes Evol.

[14] Angelini DR, Kaufman TC (2005). Functional analyses in the milkweed bug *Oncopeltus fasciatus* (Hemiptera) support a role for Wnt signaling in body segmentation but not appendage development. Dev Biol.

[15] Angelini DR, Kaufman TC (2005). Insect appendages and comparative ontogenetics. Dev Biol.

[16] Freitas R, Zhang G, Cohn MJ (2006). Evidence that mechanisms of fin development evolved in the midline of early vertebrates. Nature.

[17] Ober KA, Jockusch EL (2006). The roles of wingless and decapentaplegic in axis and appendage development in the red flour beetle *Tribolium castaneum*. Dev Biol.

[18] Kreissl S, Uber A, Harzsch S (2008). Muscle precursor cells in the developing limbs of two isopods (Crustacea, Peracarida): an immunohistochemical study using a novel monoclonal antibody against myosin heavy chain. Dev Genes Evol.

[19] Ohde T, Yaginuma T, Niimi T (2013). Insect morphological diversification through the modification of wing serial homologs. Science.

[20] Nuno Dela Rosa L, Müller GB, Metscher BD, Metscher BD (2014). The lateral mesodermal divide: an epigenetic model of the origin of paired fins. Evol Dev.

[21] Don EK, Currie PD, Cole NJ (2013). The evolutionary history of the development of the pelvic fin/hindlimb. J Anat.

[22] Moriyama Y, Takeda H (2013). Evolution and development of the homocercal caudal fin in teleosts. Dev Growth Differ.

[23] Lee PN, Callaerts P, de Couet HG, Martindale MQ (2003). Cephalopod Hox genes and the origin of morphological novelties. Nature.

[24] Farfan C, Shigeno S, Nödl MT, de Couet HG (2009). Developmental expression of *apterous/Lhx2/9* in the sepiolid squid *Euprymna scolopes* supports an ancestral role in neural development. Evol Dev.

[25] Winchell CJ, Jacobs DK (2013). Expression of the Lhx genes *apterous* and *lim1* in an errant polychaete: implications for bilaterian appendage evolution, neural development, and muscle diversification. EvoDevo.

[26] Winchell CJ, Valencia JE, Jacobs DK (2010). Expression of *Distal-less*, *dachshund*, and *optomotor blind* in *Neanthes arenaceodentata* (Annelida, Nereididae) does not support homology of appendage-forming mechanisms across the Bilateria. Dev Genes Evol.

[27] Shigeno S, Takenori S, Boletzky S, Tanabe K, Shigeta Y, Sasaki T, Hirano H (2010). The origins of cephalopod body plans: a geometrical and developmental basis for the evolution of vertebrate-like organ systems. Cephalopods - Present and Past.

[28] Boletzky S (1993). The arm crown in cephalopod development and evolution: a discussion of morphological and behavioral homologies. Amer Malacol Bull.

[29] Boletzky S (1999). Cephalopod Development And Evolution. Biological Insight into Ontogenesis as a Guide to Paleomorphology. Cephalopods: Present and Past; 1999; Granada, Spain.

[30] Shigeno S, Sasaki T, Moritaki T, Kasugai T, Vecchione M, Agata K (2008). Evolution of the cephalopod head complex by assembly of multiple molluscan body parts: evidence from *Nautilus* embryonic development. J Morph.

[31] Kier WM, Smith KK (1985). Tongues, tentacles and trunks: the biomechanics of movement in muscular-hydrostats. Zool J Linnean Soc.

[32] Kier WM, Stella MP (2007). The arrangement and function of octopus arm musculature and connective tissue. J Morph.

[33] Graziadei P, Young JZ (1971). The Nervous System of the Arms. The Anatomy of the Nervous System of *Octopus vulgaris*.

[34] Wells MJ (1964). Tactile discrimination of surface curvature and shape by the *Octopus*. J Exp Biol.

[35] Packard A (1961). Sucker display of octopus. Nature.

[36] Kier WM, Smith AM (2002). The structure and adhesive mechanism of octopus suckers. Integr Comp Biol.

[37] Semmens JM, Pecl GT, Villanueva R, Jouffre D, Sobrino I, Wood JB (2004). Understanding octopus growth: patterns, variability and physiology. Mar Freshwater Res.

[38] Lange MM (1920). On the regeneration and finer structure of the arms of the cephalopods. J Exp Biol.

[39] May RM (1933). La formation des terminaisons nerveuses dans les ventouses du bras régénéré du Céphalopode *Octopus vulgaris*. Lamm Ann Staz Océanogr Salammbô.

[40] Fossati SM, Carella F, De Vico G, Benfenati F, Zullo L (2013). Octopus arm regeneration: role of acetylcholinesterase during morphological modification. J Exp Mar Biol Ecol.

[41] Boletzky S (2003). Biology of early life stages in cephalopod molluscs. Adv Mar Biol.

[42] Naef A (1928). Cephalopoda (embryology). Fauna and flora of the Bay of Naples. Enfield (NH), USA; Plymouth.

[43] Fioroni P, Meister G (1974). *Loligo vulgaris* Lam. Gemeiner Kalmar. Groβes Zoologisches Praktikum.

[44] Marquis F (1989). Die Embryonalentwicklung des Nervensystems von *Octopus vulgaris* Lam. (Cephalopoda, Octopoda), eine histologische Analyse. Verh Naturforsch Ges Basel.

[45] Arnold JM (1965). Normal embryonic stages of the squid, *Loligo pealei* (Lesueur). Biol Bull.

[46] Fiorito G, Affuso A, Anderson D, Basil J, Bonnaud L, Botta G (2014). Cephalopods in neuroscience: regulations, research and the 3Rs. IN.

[47] Abramoff MD, Magalhaes PJ, Ram SJ (2004). Image processing with ImageJ. Biophotonics Int.

[48] Matulef K, Sirokman K, Perreault-Micale CL, Szent-Gyorgyi AG (1998). Amino-acid sequence of squid myosin heavy chain. J Muscle Res Cell M.

[49] Huelsenbeck JP, Ronquist F (2001). MRBAYES: Bayesian inference of phylogenetic trees. Bioinformatics.

[50] Lee PN, McFall-Ngai MJ, Callaerts P, de Couet HG (2009). Whole-mount *in situ* hybridization of Hawaiian bobtail squid (*Euprymna scolopes*) embryos with DIG-labeled riboprobes: II. Embryo preparation, hybridization, washes, and immunohistochemistry. Cold Spring Harb Protoc.

[51] Kölliker A (1844). Entwickelungsgeschichte der Cephalopoden.

[52] Kier WM (1996). Muscle development in squid: Ultrastructural differentiation of a specialized muscle fiber type. J Morphol.

[53] Grimaldi A, Tettamanti G, Brivio MF, Valvassori R, Eguileor M (2004). Differentiation of slow and fast fibers in tentacles of *Sepia officinalis* (Mollusca). Dev Growth Differ.

[54] Condic M, Fristrom D, Fristrom J (1991). Apical cell shape changes during *Drosophila* imaginal leg disc elongation: a novel morphogenetic mechanism. Development.

[55] Fritz AE, Ikmi A, Seidel C, Paulson A, Gibson MC (2013). Mechanisms of tentacle morphogenesis in the sea anemone *Nematostella vectensis*. Development.

[56] Tada M, Heisenberg CP (2012). Convergent extension: using collective cell migration and cell intercalation to shape embryos. Development.

[57] Weiser DC, Pyati UJ, Kimelman D (2007). Gravin regulates mesodermal cell behavior changes required for axis elongation during zebrafish gastrulation. Gene Dev.

[58] Widelitz RB, Jiang T-X, Chen C-WJ, Stott SN, Jung H-S, Chuong C-M (1999). Wnt-7a in feather morphogenesis: involvement of anterior-posterior asymmetry and proximal-distal elongation demonstrated with an in vitro reconstitution model. Development.

[59] Steinmetz PR, Kraus JE, Larroux C, Hammel JU, Amon-Hassenzahl A, Houliston E (2012). Independent evolution of striated muscles in cnidarians and bilaterians. Nature.

[60] Taylor MV (1998). Muscle development: a transcriptional pathway in myogenesis. Curr Biol.

[61] Hooper SL, Hobbs KH, Thuma JB (2008). Invertebrate muscles: thin and thick filament structure; molecular basis of contraction and its regulation, catch and asynchronous muscle. Prog Neurobiol.

[62] Carlini DB, Reece KS, Graves JE (2000). Actin gene family evolution and the phylogeny of coleoid cephalopods (Mollusca: Cephalopoda). Mol Biol Evol.

[63] Odintsova N, Dyachuk V, Kiselev K, Shelud’ko N (2006). Expression of thick filament proteins during ontogenesis of the mussel *Mytilus trossulus* (Mollusca: Bivalvia). Comp Biochem Physiol B Biochem Mol Biol.

[64] Degnan BM, Degnan MD, Morse DE (1997). Muscle-specific regulation of tropomyosin gene expression and myofibrillogenesis differs among muscle systems examined at metamorphosis of the gastropod *Haliotis rufescens*. Dev Genes Evol.

[65] Ochiai Y, Watabe S, Wang G (2013). Structural and phylogenetic profiles of muscle actins from cephalopods. J Basic Appl Sci.

[66] Motoyama K, Ishizaki S, Nagashima Y, Shiomi K (2006). Cephalopod tropomyosins: identification as major allergens and molecular cloning. Food Chem Toxicol.

[67] Fioroni P (1982). Zur Epidermis- und Saugnapfentwicklung bei Octopoden, ein entwicklungsgeschichtlicher Vergleich. Rev Suisse Zool.

[68] Nolte V, Fioroni P (1983). On the development of suckers in coleoid cephalopods. Zool Anz.

[69] Searls R, Janners M (1971). The initiation of limb bud outgrowth in the embryonic chick. Dev Biol.

[70] Benazeraf B, Francois P, Baker RE, Denans N, Little CD, Pourquie O (2010). A random cell motility gradient downstream of FGF controls elongation of an amniote embryo. Nature.

[71] Wallingford JB, Fraser SE, Harland RM (2002). Convergent extension: the molecular control of polarized cell movement during embryonic development. Dev Cell.

[72] Wyngaarden LA, Vogeli KM, Ciruna BG, Wells M, Hadjantonakis AK, Hopyan S (2010). Oriented cell motility and division underlie early limb bud morphogenesis. Development.

[73] Boehm B, Westerberg H, Lesnicar-Pucko G, Raja S, Rautschka M, Cotterell J (2010). The role of spatially controlled cell proliferation in limb bud morphogenesis. PLoS Biol.

[74] Lewandoski M, Mackem S (2011). Developmental biology: extending the limb and body with vectors and scalars. Curr Biol.

[75] Hopyan S, Sharpe J, Yang Y (2011). Budding behaviors: growth of the limb as a model of morphogenesis. Dev Dynam.

[76] Gros J, Hu JK, Vinegoni C, Feruglio PF, Weissleder R, Tabin CJ (2010). WNT5A/JNK and FGF/MAPK pathways regulate the cellular events shaping the vertebrate limb bud. Curr Biol.

[77] Topol L, Jiang X, Choi H, Garrett-Beal L, Carolan PJ, Yang Y (2003). Wnt-5a inhibits the canonical Wnt pathway by promoting GSK-3-independent beta-catenin degradation. J Cell Biol.

[78] Bradley EW, Drissi MH (2011). Wnt5b regulates mesenchymal cell aggregation and chondrocyte differentiation through the planar cell polarity pathway. J Cell Physiol.

[79] Kubota K, Goto S, Hayashi S (2003). The role of Wg signaling in the patterning of embryonic leg primordium in *Drosophila*. Dev Biol.

[80] Munder S, Tischer S, Grundhuber M, Buchels N, Bruckmeier N, Eckert S (2013). Notch-signalling is required for head regeneration and tentacle patterning in Hydra. Dev Biol.

[81] Boletzky S. Evolutionary aspects of development, life style, and reproductive mode in incirrate octopods (Mollusca, Cephalopoda). Rev Suisse Zool. 1992;99(4):755–70.

[82] Villanueva R (1995). Experimental rearing and growth of planktonic *Octopus vulgaris* from hatching to settlement. Can J Fish Aquat Sci.

[83] Buresi A, Canali E, Bonnaud L, Baratte S (2013). Delayed and asynchronous ganglionic maturation during cephalopod neurogenesis as evidenced by *Sof-elav1* expression in embryos of *Sepia officinalis* (Mollusca, Cephalopoda). J Comp Neurol.

[84] Grimaldi A, Tettamanti G, Rinaldi L, Brivio MF, Castellani D, Eguileor M (2004). Muscle differentiation in tentacles of *Sepia officinalis* (Mollusca) is regulated by muscle regulatory factors (MRF) related proteins. Dev Growth Differ.

[85] Francis-West PH, Antoni L, Anakwe K (2003). Regulation of myogenic differentiation in the developing limb bud. J Anat.

[86] Duprez D (2002). Signals regulating muscle formation in the limb during embryonic development. In J Dev Biol.

[87] Christ B, Brand-Saberi B (2002). Limb muscle development. Int J Dev Biol.

[88] Kier WM (1985). The musculature of squid arms and tentacles: ultrastructural evidence for functional differences. J Morphol.

[89] Villanueva R, Nozais C, Boletzky S (1996). Swimming behaviour and food searching in planktonic Octopus vulgaris Cuvier from hatching to settlement. J Exp Mar Biol Ecol.

[90] Stern DL, Emlen DJ (1999). The developmental basis for allometry in insects. Development.

[91] Beermann A, Aranda M, Schröder R (2004). The Sp8 zinc-finger transcription factor is involved in allometric growth of the limbs in the beetle *Tribolium castaneum*. Development.

[92] Fernandez-Teran MA, Hinchliffe JR, Ros MA (2006). Birth and death of cells in limb development: a mapping study. Dev Dynam.

