# Supplementary figures and images for: The making of an octopus arm
Source: EvoDevo. 2015 May 7;6:19. doi: 10.1186/s13227-015-0012-8 (PMC4458049; doi:10.1186/s13227-015-0012-8)

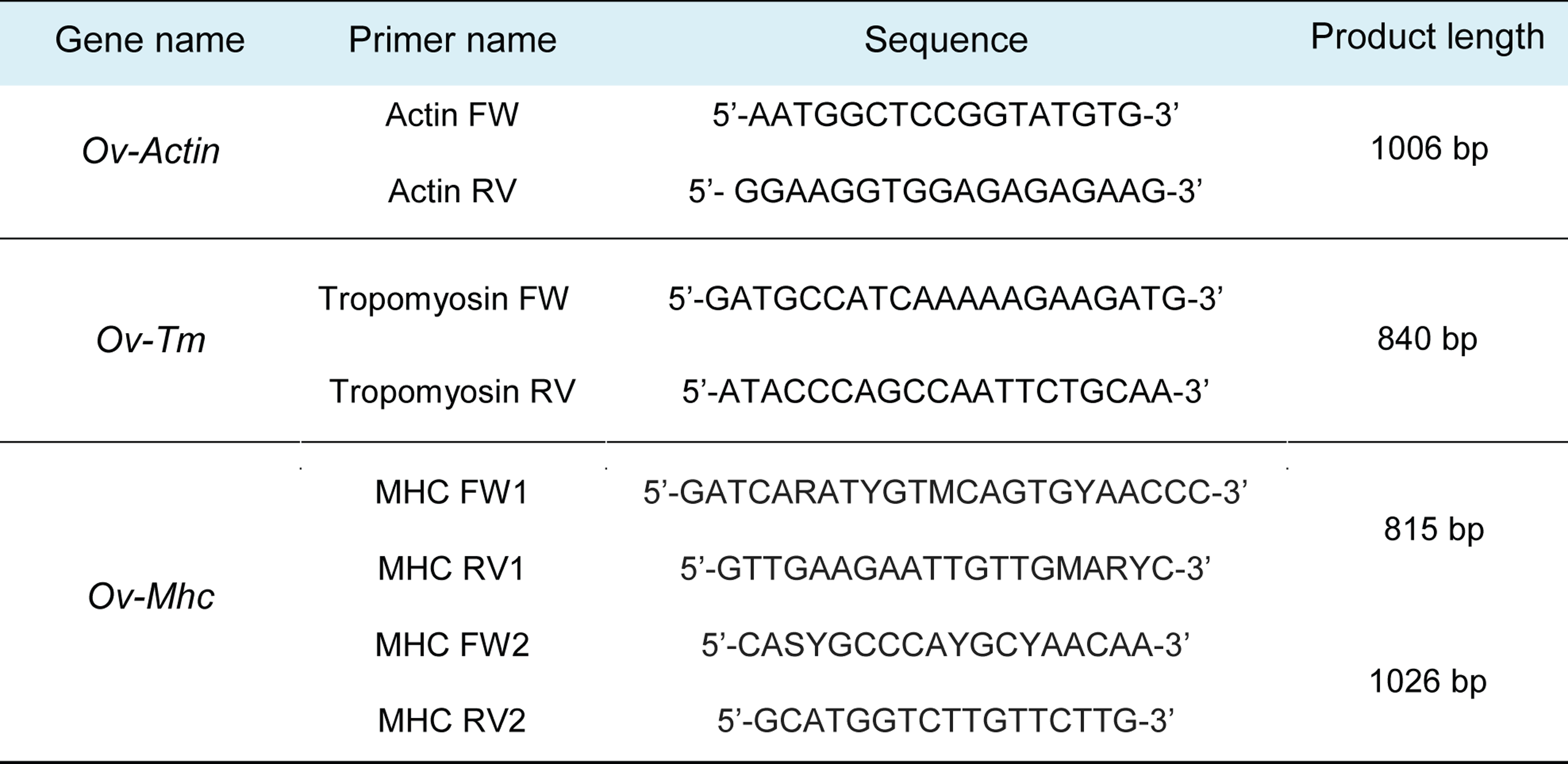

Supplement: Additional file 1: — List of specific and degenerate primers. Primers used to clone the gene fragments of Ov-Mhc, Ov-Tm, Ov-Actin, and lengths of obtained sequences. [file 13227_2015_12_MOESM1_ESM.png]

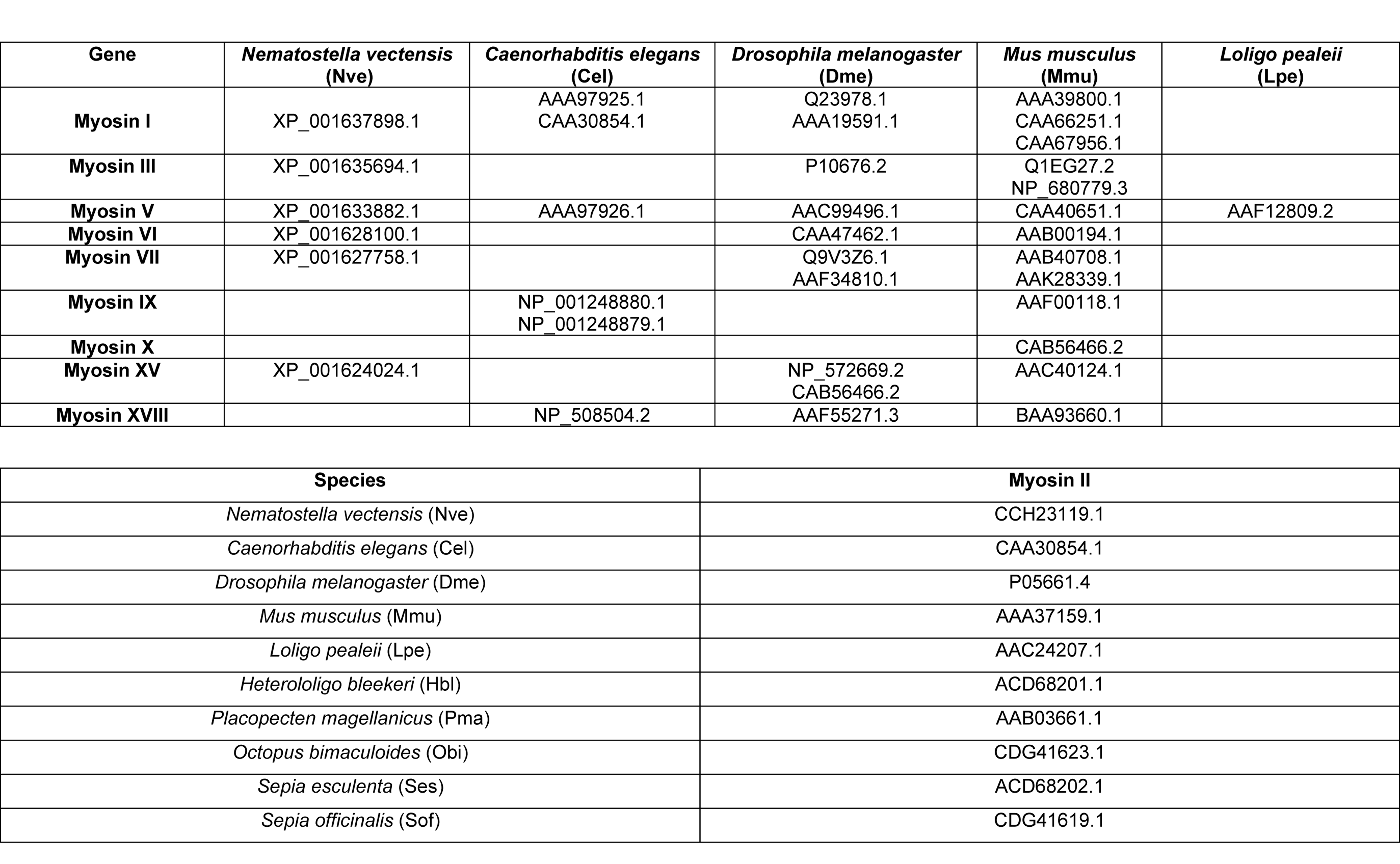

Supplement: Additional file 2: — GenBank accession numbers. List of phyla and GenBank accession numbers used in phylogenetic analysis. [file 13227_2015_12_MOESM2_ESM.png]

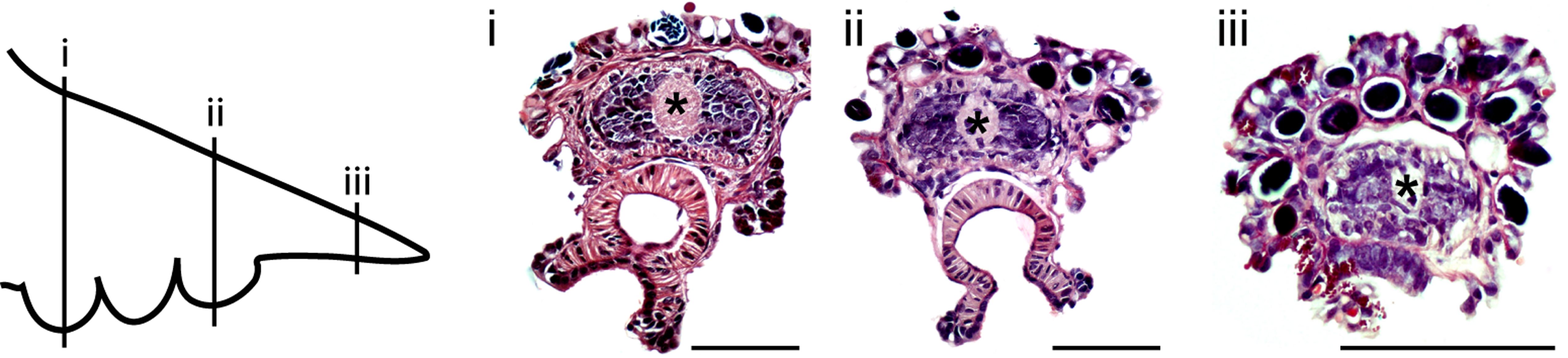

Supplement: Additional file 3: — Complexity of an octopus arm at hatching stage along its proximal-distal axis. Schematic drawing of an arm at hatching stage, which illustrates the position of the histological cross sections shown in (i, ii, iii) stained with Masson’s trichrome stain. Asterisk marks the axial nerve cord. Scale bar: 20 μm. [file 13227_2015_12_MOESM3_ESM.png]

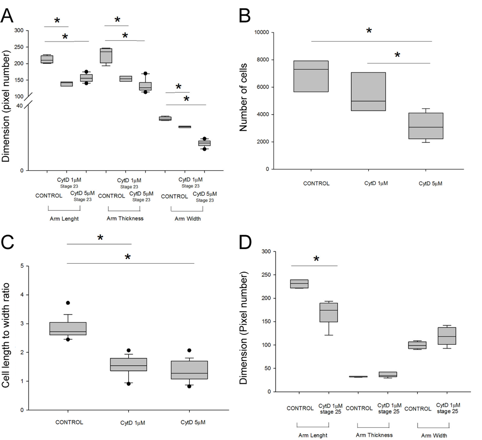

Supplement: Additional file 8: — Quantification of the morphological modifications in the arms and cells of the CytD 1 and CytD 5 treated embryos. (A) Arm length, width, and thickness of stage 23 CytD1- and CytD5-treated embryos (n = 27; ANOVA, P < 0.05). (B) Cell number of stage 23 CytD1- and CytD5-treated embryos (n = 10; ANOVA, P = 0.164 (CytD1), P < 0.05 (CytD5)). (C) Cell dimension of stage 23 CytD1- and CytD5-treated embryos (n = 60; ANOVA, P < 0.05). (D) Arm length, width, and thickness of stage 25 CytD1-treated embryos (n = 11; t test, P < 0.05). [file 13227_2015_12_MOESM8_ESM.doc]

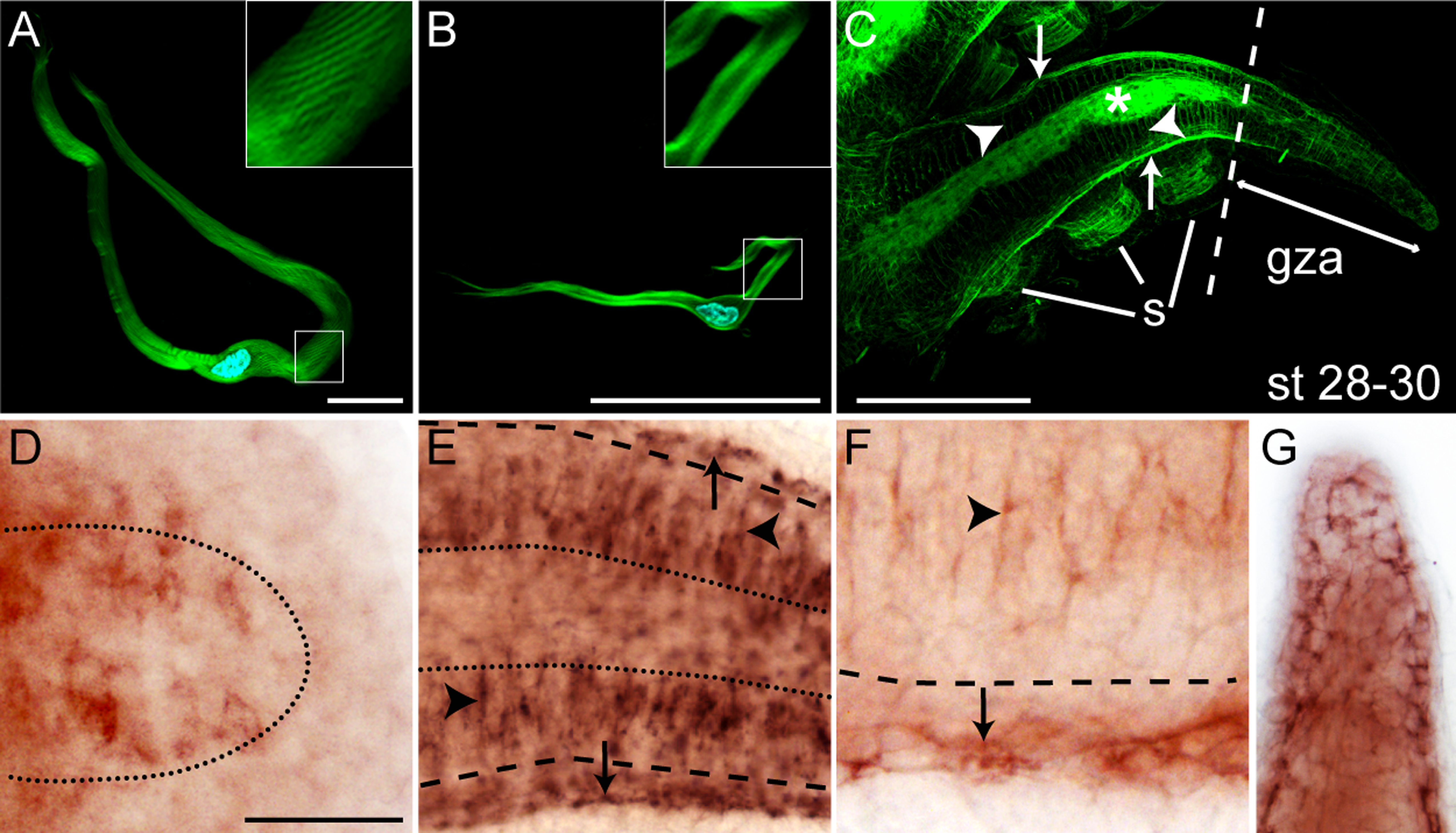

Supplement: Additional file 9: — Octopus muscle cells. Dissociated longitudinal muscle fiber of an adult animal (A) and dissociated muscle fiber of a hatchling’s arm (B) stained with Phallacidin to label F-actin (green) and Hoechst to visualize the nuclei. Insets on the upper right show a magnification of the boxed regions. Compared to the adult muscle cell, the striation in the embryonic muscle cell is not apparent yet. (C) Merged image stack of sagittal sections of individual arm stages 28 to 30 stained with phallacidin to visualize F-actin. Arrows point at longitudinal muscle layer, arrowheads denote the transverse muscle layer. Asterisk marks the area of the axial nerve cord. Dashed line indicates the area of the growth zone of the arm. (D) Close-up of stages 20 to 21 Ov-Mhc in situ hybridization. Dotted line encircles the area of neuronal precursor cells of the future axial nerve cord. (E) Close-up of stages 24 to 25 Ov-Mhc in situ hybridization. Dotted lines encircle the axial nerve cord. (F) Close-up of stage 26 Ov-Mhc in situ hybridization. Dashed line confines an area of neuronal and overlying transverse muscle cells adjacent to the axial nerve cord. (G) Close-up of the growth zone of a stage 26 arm. Abbreviation: gza, growth zone of the arm. Scale bars: A, C, D (refers to D to G): 100 μm, B: 50 μm. [file 13227_2015_12_MOESM9_ESM.png]

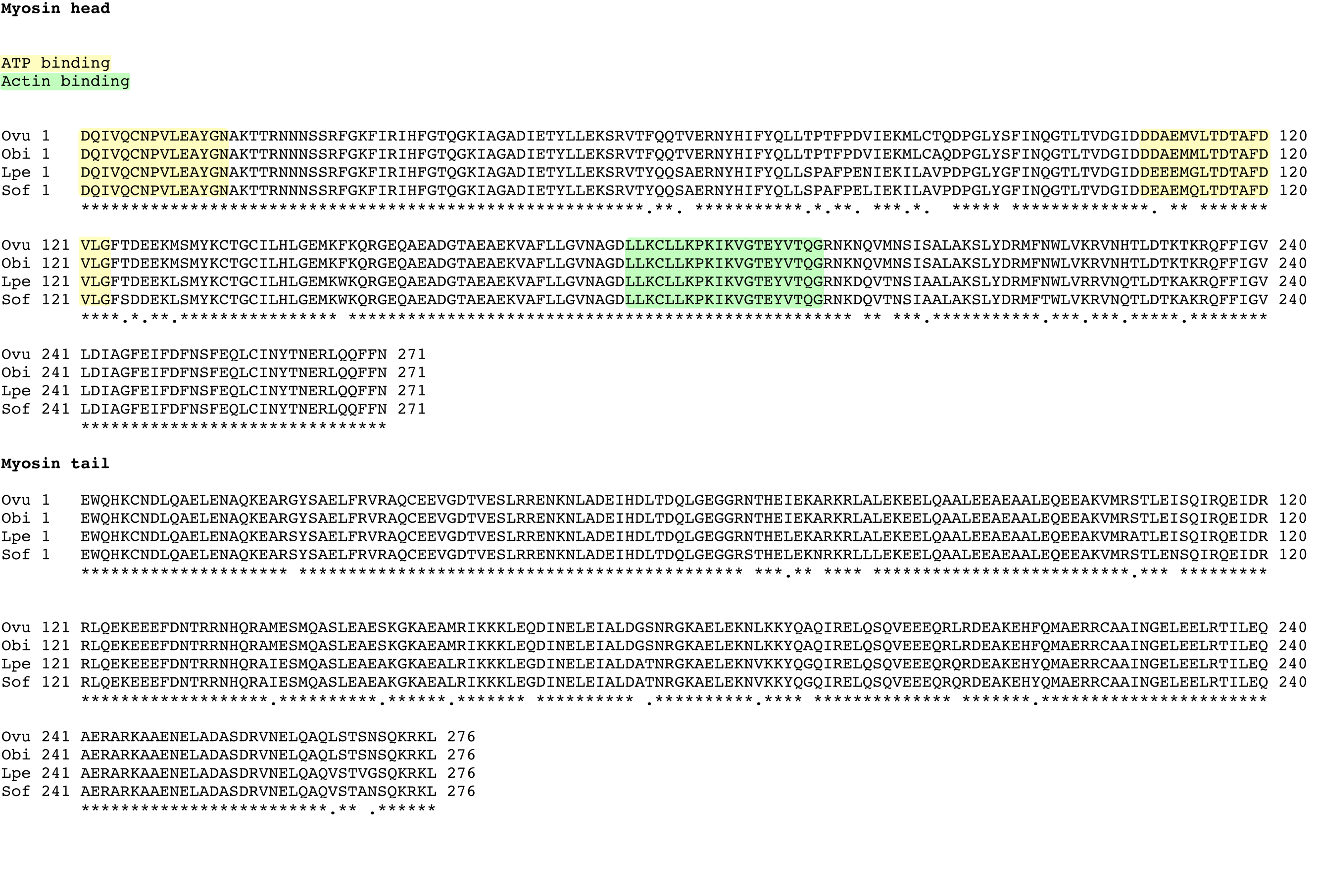

Supplement: Additional file 10: — Sequence alignment of the deduced Ov-Mhc amino acid sequences with known cephalopod orthologs. Abbreviations and accession numbers: Lpe, Loligo pealeii (AAC24207.1); Obi, Octopus bimaculoides (CDG41623.1); Ovu, Octopus vulgaris; Sof, Sepia officinalis (CDG41619.1). [file 13227_2015_12_MOESM10_ESM.png]

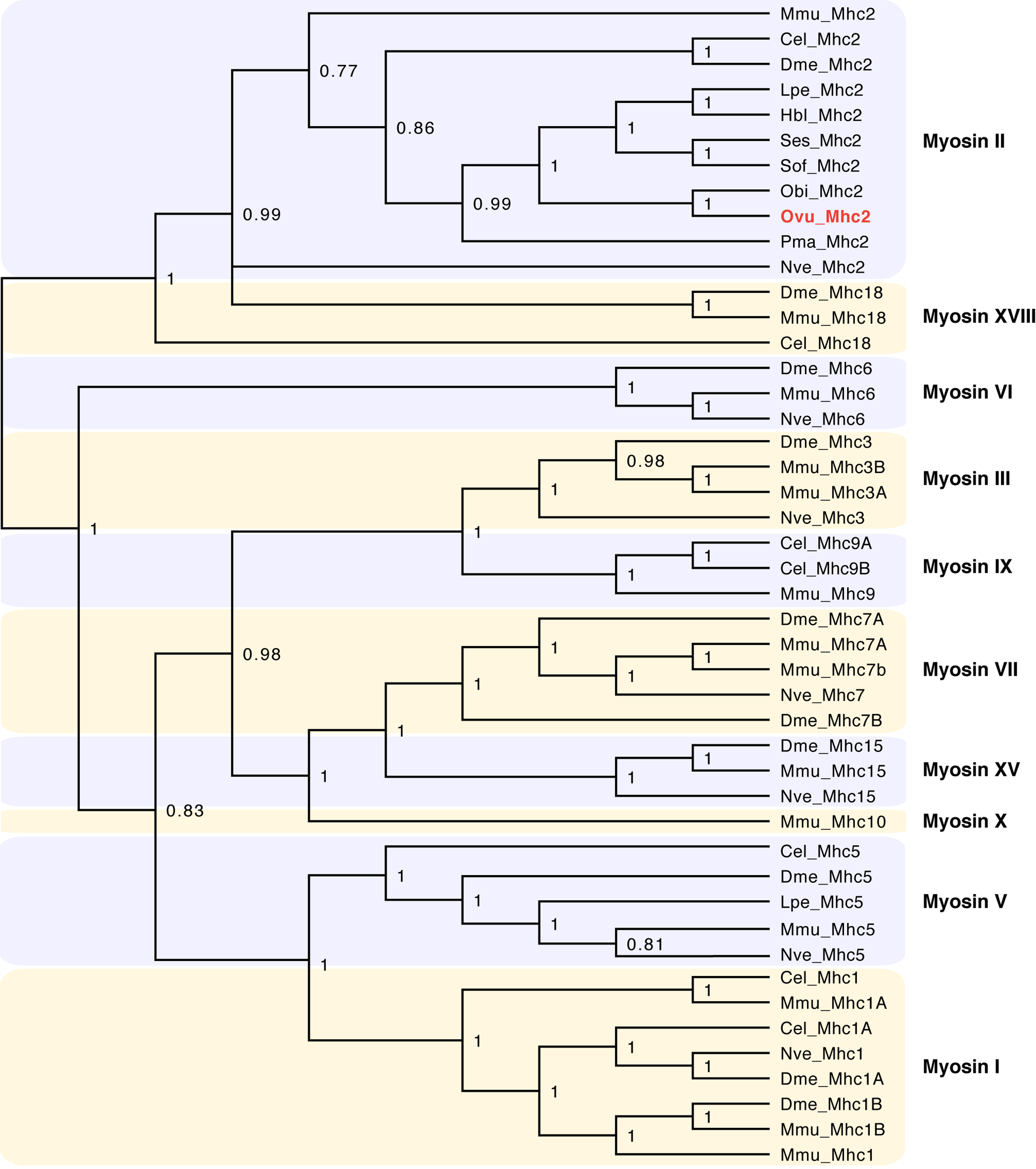

Supplement: Additional file 11: — Phylogenetic tree of the Myosin superfamily. Ov-Mhc is specifically assigned to a distinct clade of MyosinII subfamily orthologs from representatives of cnidaria, lophotrochozoa, ecdysozoa, and chordata. Numbers represent the posterior probabilities. [file 13227_2015_12_MOESM11_ESM.png]
